# Evolutionary History of LTR Retrotransposon Chromodomains in Plants

**DOI:** 10.1155/2012/874743

**Published:** 2012-04-29

**Authors:** Anton Novikov, Georgiy Smyshlyaev, Olga Novikova

**Affiliations:** ^1^Laboratory of Molecular Genetic Systems, Institute of Cytology and Genetics, Novosibirsk, 630090, Russia; ^2^Department of Natural Sciences, Novosibirsk State University, Novosibirsk, 630090, Russia; ^3^Department of Plant Pathology, University of Kentucky, Lexington, KY 40546, USA; ^4^Department of Biological Sciences, University at Albany, Life Sciences Building 2061, 1400 Washington Avenue, Albany, NY 12222, USA

## Abstract

Chromodomain-containing LTR retrotransposons are one of the most successful groups of mobile elements in plant genomes. Previously, we demonstrated that two types of chromodomains (CHDs) are carried by plant LTR retrotransposons. Chromodomains from group I (CHD_I) were detected only in Tcn1-like LTR retrotransposons from nonseed plants such as mosses (including the model moss species *Physcomitrella*) and lycophytes (the *Selaginella* species). LTR retrotransposon chromodomains from group II (CHD_II) have been described from a wide range of higher plants. In the present study, we performed computer-based mining of plant LTR retrotransposon CHDs from diverse plants with an emphasis on spike-moss *Selaginella*. Our extended comparative and phylogenetic analysis demonstrated that two types of CHDs are present only in the *Selaginella* genome, which puts this species in a unique position among plants. It appears that a transition from CHD_I to CHD_II and further diversification occurred in the evolutionary history of plant LTR retrotransposons at approximately 400 MYA and most probably was associated with the evolution of chromatin organization.

## 1. Introduction

A chromodomain (CHD) is a protein domain involved in chromatin remodeling and the regulation of gene expression in eukaryotes (e.g., [1–3]). CHDs perform a wide range of functions, including chromatin targeting and interactions between different proteins, RNA and DNA [3]. There are two major groups of CHDs that are found in eukaryotic chromodomain-containing proteins. The so-called “classical” CHDs carry the characteristic chromo-box motif (Y/f)-(L/F/Y)-(L/I/V)-K-(W/y)-(k/r)-g (single-letter code, capital letters standing for the most prominent aminoacid) [4]. The “classical” CHDs are highly conserved among eukaryotes and are represented in a large number of proteins in many genomes. They are believed to have a similar three-dimensional structure, which consists of an N-terminal three-stranded *β*-barrel capped by a C-terminal helix [5]. Three conserved residues, Y24, W45, and Y48, are essential for aromatic pocket formation [6, 7].

The second group of CHDs, “shadow” chromodomains, is more variable and includes chromo-related domains, which are well conserved in their central region, but they deviate significantly in other regions. The majority of the “shadow” CHDs contain the conserved residue W45 and lack Y24 and Y48 [3, 4]. In comparison with the “classical” CHDs, the shadow chromodomains contain one helix at the N-terminus and another inserted before the C-terminal helix [8]. The best-known protein with both types of chromodomains is heterochromatin protein-1 (HP1). HP1 presence is a hallmark of constitutive heterochromatin in *Drosophila*, a condensed and highly repressive type of chromatin that organizes the repetitive pericentromeric DNA. This protein contains an N-terminal “classical” CHD and a C-terminal shadow chromo-related domain. The CHD of HP1 binds to histone H3 dimethyl-K9 (H3K9me2) and histone H3 trimethyl-K9 (H3K9me3) to help establish transcriptionally silent heterochromatin [9–11].

The chromodomain has been found not only in eukaryotic functional proteins but also in diverse LTR retrotransposons, which are called chromodomain-containing Gypsy LTR retrotransposons or chromoviruses [12, 13]. Chromoviruses are the most widespread lineage of Gypsy LTR retrotransposons and are present in the genomes of fungi, plants, and vertebrates [13, 14]. Two distinct groups of retrotransposon CHDs have been described. Group I CHDs from retrotransposons (CHDs_I) are similar to “classical” CHDs of chromodomain-containing proteins [15]. This group of CHDs was found in diverse eukaryotic LTR retrotransposons, including fungal and vertebrate Gypsy elements, as well as in LTR retrotransposons from moss *Physcomitrella patens* and spike-moss *Selaginella moellendorffii*, which belong to the Tcn1 clade [13, 16–18]. The information on the role of CHDs_I in the retrotransposition of LTR retrotransposons is limited. The transposition activity of the MAGGY retrotransposon of the rice blast fungus *Magnaporthe oryzae *dramatically decreased with the loss or alteration of the chromodomain [19]. On the other hand, the chromointegrase of the Tf1 LTR retrotransposon from *Schizosaccharomyces pombe *that lacks the chromodomain demonstrated a significantly higher activity and a substantially reduced substrate specificity [20]. As was demonstrated recently, the MAGGY chromodomain interacts with H3K9me2 and H3K9me3 in a similar way compared to the HP1 “classical” chromodomain. It was proposed that chromodomains can target the integration of chromoviruses into heterochromatic regions [15].

Representatives of group II CHDs from retrotransposons (CHDs_II) lack the first conserved aromatic residue (Y24) and usually the third (Y48). Group II has only been identified in plant Gypsy LTR retrotransposons. Little is known about the activity and role of CHDs_II from plant retrotransposons. The mostly heterochromatic distribution of plant chromoviruses along with data describing the localization of chromodomain-YFP fused protein in heterochromatin can be used as indirect evidence for recognizing heterochromatin and directing the integration role of CHDs_II [15]. Nevertheless, the actual mechanisms with which these chromodomains act are still unknown.

Previously, we demonstrated that our knowledge of plant chromodomain-containing LTR retrotransposons is mostly limited to knowledge about seed plants (mostly angiosperms) [16–18]. An investigation of retrotransposons from nonseed plants could shed light on the evolutionary history of retrotransposons and their impact on the evolutionary history of plant genomes. For example, it is still not clear whether CHDs_I and CHDs_II were acquired independently by distinct lineages of LTR retrotransposons or whether they evolved from a common ancestor. The present survey of chromodomains from diverse plants with an emphasis on the spike-moss *Selaginella* demonstrated that a transition from CHDs_I to CHDs_II occurred in the evolutionary history of plants approximately 500–400 MYA. Moreover, several types of clade-specific CHDs_II were found in plants; sequence dissimilarities among these clade-specific CHDs hypothesized to indicate functional differences. We examined the evolutionary constraints that shaped the diversity of CHDs_II in plants and demonstrated that positive selection contributed to the diversification of clade-specific LTR retrotransposon CHDs. We propose that the presence of CHDs_I or CHDs_II is related to the distribution of heterochromatin/euchromatin marks and molecular differences in these marks between distinct lineages of eukaryotes, such as fungi/metazoa and plants. Both the transition from CHDs_I to CHDs_II and the diversification of clade-specific CHDs reflect evolutionary changes that occurred in plant chromatin organization.

## 2. Results

### 2.1. Novel LTR Retrotransposons from *Selaginella moellendorffii*

**Figure 1:**
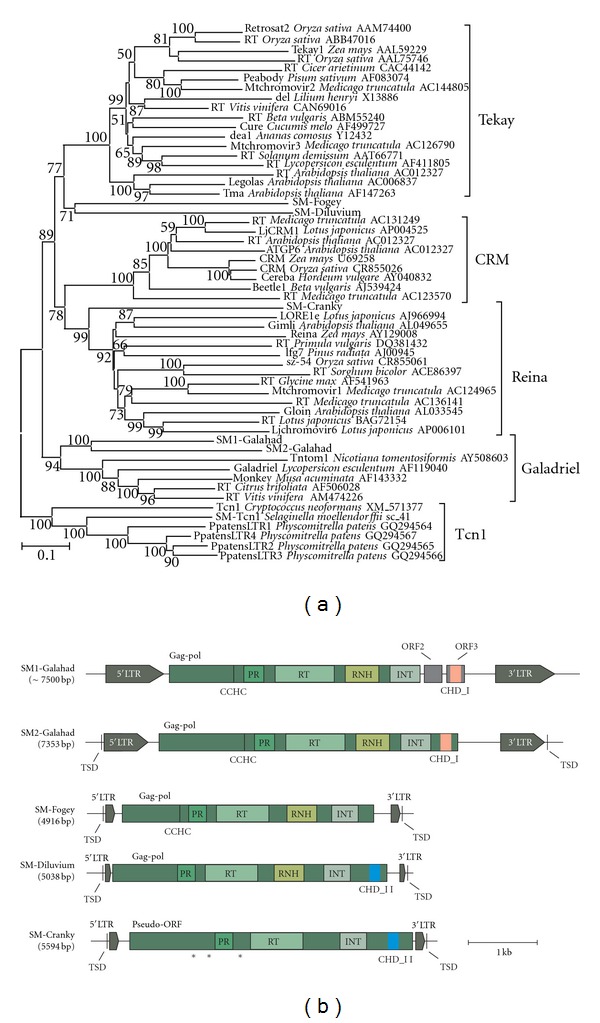
A neighbor-joining (NJ) phylogenetic tree based on multiple alignment of reverse transcriptase aminoacid sequences of LTR retrotransposons, including newly identified chromodomain-containing LTR retrotransposons from *Selaginella moellendorffii* (a) and the structural organization of novel retroelements from *Selaginella* (b). Statistical support was evaluated by bootstrapping (1000 replications); nodes with bootstrap values over 50% are shown. The plant-specific clades: Reina, CRM, Galadriel, and Tekay, as well as the Tcn1 clade, are indicated. The name of the host species and the accession number is indicated for the LTR elements that were taken from GenBank. Abbreviations: ORF: open reading frame, PR: aspartyl protease, RT: reverse transcriptase, RNH: ribonuclease H, INT: integrase, CHD_I and CHD_II: chromodomain group I and group II, CCHC: Zn-finger motif, TSD: target site duplications, and 5′ and 3′LTRs: 5′ and 3′ long terminal repeats. The positions of stop-codons (SM-Cranky) are marked by asterisks.

Previously, we described SM-Tcn1 CHD-containing Gypsy LTR retrotransposons, which presumably appeared as a result of a horizontal transfer from fungi during the early evolution of plants [18]. Several other families of LTR retroelements were identified by performing BLASTN and TBLASTN searches of the *S. moellendorffii* Whole Genome Shotgun (WGS) database (http://genome.jgi-psf.org/Selmo1) using the SM-Tcn1 retrotransposon as well as previously described retrotransposons from other species as queries (see Section 4). The newly identified retrotransposons were classified as representatives of the same or different families based on the levels of their similarities. More than 80% identity at the nucleotide level is believed to be sufficient for the classification of retrotransposons in the same family [21]. An additional criterion to designate retrotransposon families is a minimum of 50% nucleotide identity in LTRs [22]. The exemplar element was retrieved or reconstructed based on copies that were available for each family. These retrotransposons were used for further classification based on comparative and phylogenetic analysis, which included known LTR retrotransposons from other plants. The result of this analysis is shown in Figure 1. In total, five diverse families of CHD-containing LTR retrotransposons were found in addition to the previously described SM-Tcn1 [18]. The five families were named SM1-Galahad and SM2-Galahad, SM-Fogey, SM-Diluvium, and SM-Cranky (Table 1).

SM1-Galahad and SM2-Galahad are closely related to each other and form a common clade with Galadriel-like retrotransposons from monocots and dicots, suggesting that this clade originated before nonvascular and vascular plants separated from a common ancestor, roughly 400 MYA [23]. Among the sequences that are available in the NCBI protein database based on BLASTP analysis, the retroelement that is most closely related to SM1-Galahad and SM2-Galahad is the Galadriel LTR retrotransposon from *Lycopersicon esculentum*, which was identified in the Cf-9 disease resistance gene cluster [24].

We did not identify a putative intact copy of SM1-Galahad in* S. moellendorffii* WGS; thus, we used a number of copies to obtain a consensus sequence. SM1-Galahad is highly repeated and represented by hundreds of copies per genome, which share 95% nucleotide similarity on average. Many copies contain large deletions and/or insertions. The reconstructed SM1-Galahad is 7.5 kbp in length and carries LTRs that are more than 1 kbp in length and which possess a short inverted terminal repeat (TG*⋯*CA), typical for LTR retrotransposons and retroviruses [25]. They also contain polyguanine tracks that vary in length (from 11 to 25 bp) between different copies. A 13 bp primer binding site-like sequence (PBS) that is complementary to the 3′ end region of tRNA^Met^ is present downstream of the 5′LTR, and a polypurine tract (PPT) was detected immediately upstream of the 3′LTR (Figure 1(b)). The putative single open reading frame (ORF) of SM1-Galahad is 3885 bp in length. The hypothetical protein product of the ORF of SM1-Galahad (1295 aa) exhibits significant similarity to both Gag and Pol gene products of known retroelements, especially with those of Gypsy retrotransposons. A search for characteristic motifs within the polyprotein sequence identified several functional domains, such as the cysteine or Zn-finger motif with Cys-X_2_-Cys-X_4_-His-X_4_-Cys (CCHC) composition, proteinase (PR), reverse transcriptase (RT), ribonuclease H (RNH), and core integrase (core Int), in the order indicated, confirming that SM1-Galahad belongs to the Ty3/Gypsy LTR retrotransposons. No chromodomain (CHD) was found at the C-terminal end of the Gag-Pol protein from the reconstructed SM1-Galahad. However, analysis of the DNA sequence downstream of the putative ORF revealed two additional short ORFs (316 bp and 309 bp in length). The 309-bp ORF3 encodes a protein (113 aa) containing a CHD group I (CHD_I) domain.

The full-length putatively intact SM2-Galahad was found in scaffold_24 (between position 1030224 and 1037576). This LTR retrotransposon is represented by hundreds of copies per genome. SM2-Galahad is a 7.4 kbp LTR retrotransposon with a single ORF (Figure 1(b)). LTRs are 919 bp in length and share 99.7% similarity. A 5 bp target site duplication (TSD) was also detected (CCTAT*⋯*CCTAT). An 11 bp PBS complementary to the tRNA^Met^ is located just downstream of the 5′LTR, and 13 bp PPT is located upstream of 3′LTR. The putative ORF (4731 bp) encodes a fused Gag-Pol protein (1577 aa), which carries several functional domains, including the CCHC Zn-finger motif, PR, RT, RNH, core Int, and CHD_I.

The complete retrotransposons SM-Fogey and SM-Diluvium are 4.9 kbp and 5 kbp long, respectively (Figure 1(b)). The full-length intact copies of SM-Fogey and SM-Diluvium can be found in scaffold_20 (position from 2127930 to 2132846) and scaffold_73 (position from 936919 to 941957). Target site duplications of five base pairs mark the integration of the intact SM-Fogey and SM-Diluvium in the genomic sequence. Both LTR retrotransposons contain four catalytic regions, PR, RT, RNH, and Int of the retroviral genes *gag* and *pol*, in a continuous single open reading frame. SM-Diluvium also has a CHD group II (CHD_II), whereas SM-Fogey does not carry a chromodomain. SM-Fogey is characterized by 233 bp terminal repeats; in contrast, the LTRs of SM-Diluvium are only 112 bp in length. The LTRs of SM-Fogey and SM-Diluvium contain consensus short inverted terminal repeats (TG*⋯*CA), which are important for the integration of retroviral sequences [26]. The primer binding site (PBS), which is complementary to the 3′ end of tRNA^Met^ and has a one-nucleotide spacer next to the adjacent 5′ LTR, was identified in both retrotransposons. The PPT with the stretch of 14 purines for SM-Fogey and SM-Diluvium is located immediately before the 3′ LTR. Several full-length highly similar copies for both SM-Fogey and SM-Diluvium can be found in* S. moellendorffii * WGS. For example, nine putatively intact copies of SM-Fogey presented in WGS share 98.5% average similarity at the DNA level. All of them are flanked by 5-bp TSD. Despite the fact that only one full-length copy was detected for SM-Diluvium, seven full-length copies presented in the genome showed an average 99.1% DNA similarity. Additionally, the 5′ and 3′LTRs of elements have high similarity, 98.7% on average for SM-Fogey and 97.3% on average for SM-Diluvium. Altogether, these features indicate that SM-Fogey and SM-Diluvium recently retrotransposed and may still be active.

The putative Gag-Pol polyproteins of SM-Fogey and SM-Diluvium were compared to those reported for other plant LTR retrotransposons. The aminoacid domains show the highest similarity to the corresponding regions of the Tekay-like retrotransposon SHMIDT that is adjacent to the disease resistance-priming gene NPR1 in *Beta vulgaris* (EF101866; [27]) and retrotransposons from the diverse *Oryza* species, including LTR retrotransposons from *Oryza sativa* and *Oryza australiensis* (e.g., DQ365821, DP000086; [28]). It appears that SM-Fogey and SM-Diluvium are the most closely related to the Tekay clade of CHD-containing Gypsy LTR retrotransposons from plants. They formed a common branch on the phylogenetic tree, but neither SM-Fogey nor SM-Diluvium can be assigned to this clade because of the low bootstrap support for this cluster (bootstrap 77%; Figure 1(a)).

One more CHD-containing Gypsy LTR retrotransposon, SM-Cranky, was found to be present with a few copies per genome. We detected only one full-length SM-Cranky located in scaffold_1 (between position 5651434 and 5657027) and six additional truncated copies. The retrotransposon is 5.6 kbp long with LTRs of 217 bp, terminating in the two-nucleotide inverted repeat (TG*⋯*CA). The LTRs are 98.6% identical to each other. A stretch of 12 bp located with two-nucleotide spacers downstream of the 5′LTR is complementary to the 3′ end of tRNA^Met^, probably providing a primer site for reverse transcription. The 14-bp PPT was found upstream of the 3′LTR. The putative pseudo-ORF is interrupted by three stop codons, but there are no frameshifts. This sequence can be translated to the protein that bears a resemblance to the characteristic PR, RT, Int, and CHD_II motifs (Figure 1(b)).

### 2.2. Several Types of Retrotransposon Chromodomains in Plants

The most intriguing finding that concerns the CHD-containing Gypsy LTR retrotransposons from the spike-moss *S. moellendorffii* is the presence of both types of LTR retrotransposon chromodomains, CHD_I and CHD_II, in the same plant genome. The SM-Tcn1 [18], SM1-Galahad, and SM2-Galahad LTR retrotransposons from *Selaginella* carry CHD_I, while SM-Diluvium and SM-Cranky contain CHD_II. The distribution of both retrotransposon CHD types among different taxa is considered to be well known. CHD_I is typical for fungal and animal LTR retrotransposons as well as for green algae (Chlamyvir clade), whereas only CHD_II was found in LTR retrotransposons from seed plants (Tekay, Galadriel, and Reina clades [13]). However, it appears that phylogenetic distribution of CHD_I-containing elements extends to most if not all extant green plant lineages. Previously, we reported that CHD_I-containing LTR retrotransposons belonging to the Tcn1 clade can be found in both moss *Physcomitrella *and spike-moss *Selaginella *[16, 18].

To expand our understanding of the evolution and diversity of retrotransposon chromodomains in green plants, we implemented a search throughout sequence databases including but not limited to PlantGDB (http://www.plantgdb.org/) and Phytozome (http://www.phytozome.net/). A plant retrotransposon CHD search was performed with (TBLASTN) using aminoacid sequences of known CHDs as queries (see Section 4). Altogether 114 plant species were investigated and results for some of them are presented in Table 2. The full list and primary information can be found in Supporting Information Table 1S and Table 2S. It should be noted that the majority of sequences available were derived from EST databases. In fact, CHD-containing retrotransposons are barely expressed, and as a rule, they are underrepresented in ESTs [13, 16]. Another factor that has an effect on the final result is the presence of a strong bias with respect to the species diversity that is represented in databases. More than 83% of the analyzed species (95 out of a total of 114) were angiosperms, and only a few representatives from other green plant groups are currently available. Among the analyzed species, only 26 did not produce any significant hits. For 80 species, retrotransposon CHDs were detected in ESTs; CHDs were also found in GenomeSurvey Sequences (GSS) and Whole Genomic Sequences (WGS) databases for 46 investigated species. Among those species for which the WGS database is available, a few did not show the presence of retrotransposon CHDs, including red algae (*Porphyrayezoensis*, *Galdieriasulphuraria*, and *Cyanidioschyzonmerolae*) as well as one green algae, *Micromonas pusilla *CCMP1545. This result can arise from either the limited number of sources of sequences available or the loss of this type of retrotransposon.

The majority of the CHDs detected belonged to group II. Only the representatives of the Chlamyvir clade showed the presence of CHD_I which was found in green algae *Chlorella vulgaris *C-169 and *Chlamydomonas reinhardtii *(Table 2; [13]). The source of ESTs as well as genomic sequences of gymnosperms is very limited, especially in comparison to those for angiosperms. Nevertheless, we were able to identify a few LTR retrotransposon CHDs in all of the species investigated (Table 2; Supporting Information Table 1S).

**Figure 2:**
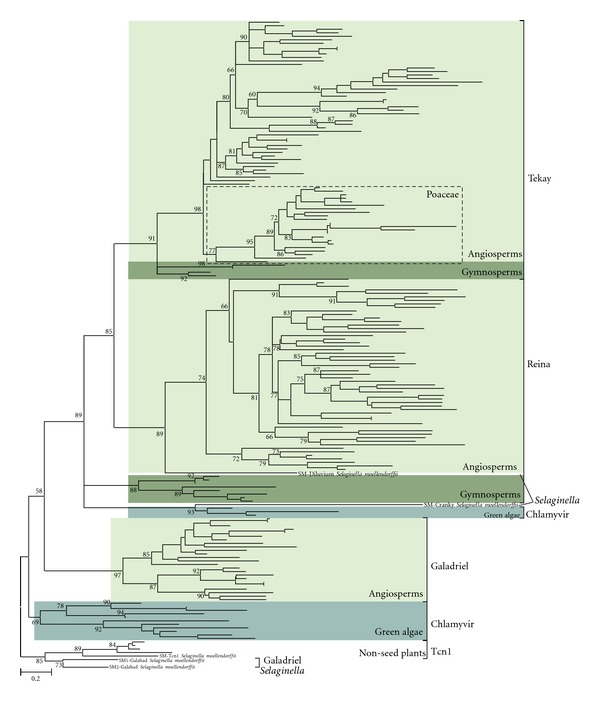
Maximum likelihood (ML) phylogenetic tree based on LTR retrotransposon chromodomain (CHD) aminoacid sequences including CHDs from newly identified chromodomain-containing LTR retrotransposons from *Selaginella moellendorffii*. Statistical support was evaluated by using aLTR; nodes with aLTR statistics over 50% are shown. The plant-specific clades Chlamyvir, Reina, CRM, Galadriel, and Tekay, as well as the Tcn1 clade, are indicated. The names of the host species and the accession numbers for the LTR elements are available in Supporting Information Figure 1S. The taxonomic range of the host species is indicated by colored boxes and includes angiosperms, gymnosperms, green algae, and nonseed plants (*Selaginella* and *Physcomitrella*).

Comparative analysis indicated that almost all of the chromodomains of type II can be easily classified as Reina-, Tekay-, or Galadriel-like CHDs based on their sequence similarity. Almost all of the plant species that produced hits in our search contain Reina- and Tekay-like CHDs. Galadriel-like CHDs were underrepresented in the analyzed databases. The phylogenetic analysis based on the aminoacid sequences of newly identified CHDs and CHDs from known plant LTR retrotransposons support these findings with several exceptions: (i) Galadriel-like LTR retrotransposons from *Selaginella* have CHD_I and are grouped with the Tcn1 clade; (ii) CHDs from conifers, which were previously believed to belong to Reina-like LTR retrotransposons from angiosperms, actually form their own branch; (iii) three CHDs identified in green algae grouped together with the *Selaginella* SM-Cranky LTR retrotransposon and not with CHDs from the Chlamyvir clade (Figure 2; [13]). Additionally, Tekay-like LTR retrotransposon CHDs from gymnosperms formed a common cluster that appears to be distinct from other Tekay-like CHDs. It is worthwhile to note that Tekay-like CHDs retrieved from representatives of the family Poaceae formed their own branch, with fairly high bootstrap support (bootstrap value 77%; Figure 2). Tekay-like LTR retrotransposons are known to be highly repeated in grass genomes. Moreover, retrotransposon activity has been implicated as playing a major role in genome size evolution in angiosperm lineage. This has been especially well-characterized in the Poaceae (e.g., [29, 30]).

### 2.3. Clade-Characteristic Protein Motifs Can Be Found in Plant CHDs

**Figure 3:**
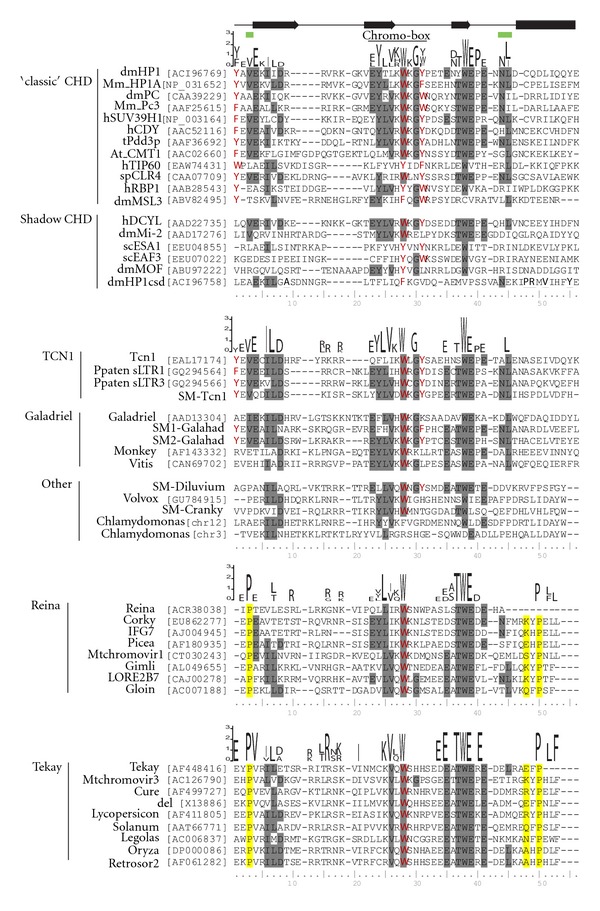
Multiple alignment of chromodomains. The following were used in the alignment: the “classical” and “shadow” chromo-like motifs of functional proteins from diverse animal species and *Arabidopsis* (At_CMT1); group I and group II chromodomains from diverse plant LTR retrotransposons belonged to Tcn1, Galadriel, Reina, and Tekay clades as well as unclassified (Other); CHD_I from Tcn1 LTR retrotransposons of fungi *Cryptococcus neoformans*. The GenBank accession numbers are indicated for each sequence. The most conservative domains for each of the groups are shown on the top. The chromo-box is indicated on the very top, which also shows the secondary structure elements (arrows indicate *β*-strands; rectangle *α*-helix) and conserved residues that form the complementary surface that is responsible for H3 peptide recognition (green boxes) [3–7]. Three aminoacids that have been shown to be under positive selection are highlighted in yellow.

Comparative analysis of primary sequences and tertiary structures of retrotransposon CHDs and the CHDs from functional proteins with known function can provide important insights into the possible roles of some of the specific aminoacids and the retrotransposon CHDs as a whole [3, 31–33]. Based on multiple alignments of selected CHDs from different clades as well as “classical” and shadow CHDs from cellular functional proteins, we identified changes in plant CHDs starting with the CHD_I found in LTR retrotransposons from green algae, moss *Physcomitrella,* and spike moss *Selaginella *and culminating in the CHD_II that is isolated from genomes of gymnosperms and angiosperms (Figure 3). The CHDs of LTR retrotransposons obtained from the *Selaginella* genome represent transitional stages between CHD_I and CHD_II. For example, SM2-Galahad CHD is close to the “classical” CHDs, whereas SM1-Galahad CHD contains a few substitutions in the chromo box motif (Y/f)-(L/F/Y)-(L/I/V)-K-(W/y)-(k/r)-g. The chromo-box is one of the motifs that is essential for hydrophobic core formation [31, 32]. This motif is characteristic for the “classical” CHDs of chromodomain-containing proteins [3]. SM-Diluvium, lacks Y24 (corresponding to position 1 in the multiple alignment represented in Figure 3), but it still has both aromatic aminoacids, W45 and Y48 (positions 28 and 31), which form methyl binding cages [3, 32].

With respect to different clades of LTR retrotransposons, Galadriel-like CHDs lost essential aminoacids (Y24 and Y48) and diverged significantly from CHD_I. Nevertheless, Galadriel-like CHDs have the conservative motif (Y/f)-(L/Y)-(I/V)-k-W-k-g (single-letter code, capital letters represent the most prominent aminoacid), which is very close to the chromo box. Reina-like LTR retrotransposons have traces of this motif, but Tekay-like CHDs have lost the motif with the exception of V43 (position 26 in the multiple alignment) and the highly conservative residue W45 (Figure 3). At the same time, Tekay-like CHDs have a number of conservative characteristic motifs. For example, the protein motif (K/R)-X-(L/T)-R-X-(k/r) is present in all of the investigated Tekay-like retrotransposon CHDs but not in Reina- or Galadriel-like CHDs. One more Tekay-characteristic motif, EEXTWEXE, is highly conserved in Tekay CHDs. A similar motif can be found in diverse LTR retrotransposon CHDs; however, this motif is not as conserved in other clades as it is in Tekay. The aminoacid motif TWE is extremely conserved among all of the retrotransposon CHDs, with a few exceptions in green algae and is believed to have important functions. Interestingly, this particular motif is absent in the majority of shadow CHDs and in a few “classical” CHDs of chromodomain-containing proteins. This motif corresponds to the *β*3 strand in the tertiary structure [3, 31].

While a majority of plant retrotransposon CHDs lack conserved aromatic residues Y24 and Y48, they still retain high sequence similarity with known “classical” CHDs of functional proteins. This similarity is much higher than the similarity among “classical” and shadow CHDs. Moreover, the analysis of tertiary structure shows the presence of all of the structural features that are characteristic of “classical” CHDs (see below).

### 2.4. Positive Selection for Retrotransposon CHDs in Plants

For further understanding of processes that lead to the current diversity of plant retrotransposon CHDs, we examined evolutionary constraints that shape the essential domains of plant LTR retrotransposon polyproteins. The presence of conservative motifs in CHD sequences among diverse LTR retrotransposons suggests that evolution has been strongly constrained. At the same time, the presence of conserved clade-specific motifs, as well as the transition from CHD_I to CHD_II can indicate that some aminoacid changes had a selective advantage during diversification between clades and could be accumulated at a rate higher than expected under natural evolution (positive selection). To distinguish between these possibilities and to indicate the positions that evolved under positive selection, we analyzed the nonsynonymous to synonymous substitutions rate ratio (*ω*, see Section 4 for details).

**Figure 4:**
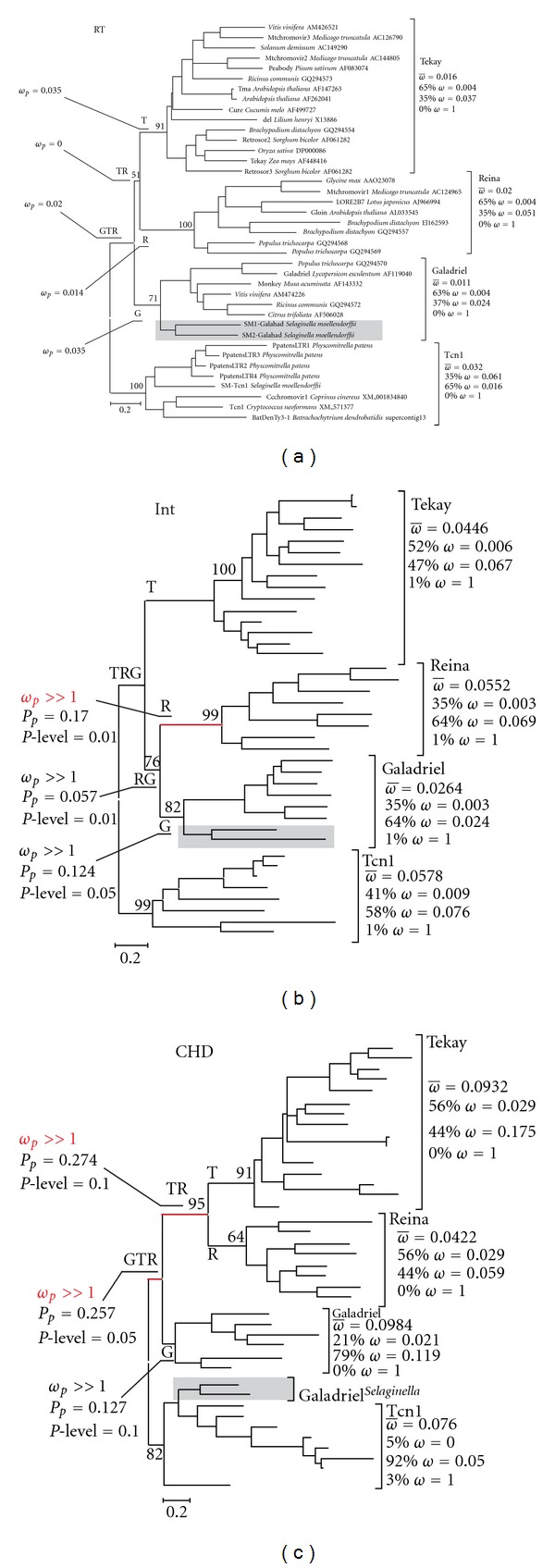
Maximum likelihood (ML) phylogenetic trees of sampled reverse transcriptase (a), integrase (b), and chromodomains (c) from 39 LTR retrotransposons. The plant-specific clades: Reina, Galadriel, and Tekay, as well as the Tcn1 clade, are indicated. Statistical support was evaluated by bootstrapping (100 replications); bootstrap values within clades are not shown. The changing in position of SM1-Galahad and SM2-Galahad LTR retrotransposons from *Selaginella *are shown by a gray box for each tree. The results of selection tests are reported for the tested branches, as is the proportion of sites under particular selective regimes for clades/groups. The red color indicates branches where sites under positive selection at the cutoff posterior probability 90% were identified.

Only retrotransposons that maintained intact domains were chosen for further analysis. CHD, core integrase (Int), and reverse transcriptase (RT) domains of 39 LTR retrotransposons were used as datasets. Phylogenetic trees based on multiple alignments of nucleotide sequences were reconstructed for each domain separately. As expected, the major difference in tree topologies among datasets included the appearance of common clusters for Galadriel-like LTR retrotransposons from *Selaginella* and the Tcn1 clade on tree reconstructions based on CHD sequences. Overall, RT- and CHD-based phylogenies are similar, whereas the Int-based tree has a different position for the Reina clade (Figure 4).

First, we estimated the *ω* ratio averaged over all of the sites and all of the lineages using M0 model. This model yields estimates that are close to 0: $\hat{\omega }=0.022$ for RT, $\hat{\omega }=\;0.039$ for Int, and $\hat{\omega }=0.068$ for the CHD domain. This low rate indicated that there is a dominating role of purifying selection in the evolution of all of the domains. The clade-site test demonstrated that no events of positive selection were inferred for RT, which was expected for a highly conserved protein domain evolving under strict constraints (e.g., [34–36]). Unexpectedly, strong positive selection was detected by clade-site test on the branch subtending the Reina clade on the Int phylogenetic tree (R branch; Figure 4(b)). This result was confirmed by a branch-site test of positive selection (modified model A—modified model A with *ω*_2_ = 1 fixed comparison) that detected positive selection on the R branch with a significance level of 0.01 (Table 3), taking into consideration multiple testing corrections (see Section 4 for details). Inference of positive selection can be an artifact when the synonymous substitutions reaching saturation. However, it is highly unlikely, taken in consideration the nature of the analyzed sequences and the pattern of substitutions (see Figure 3).

**Figure 5:**
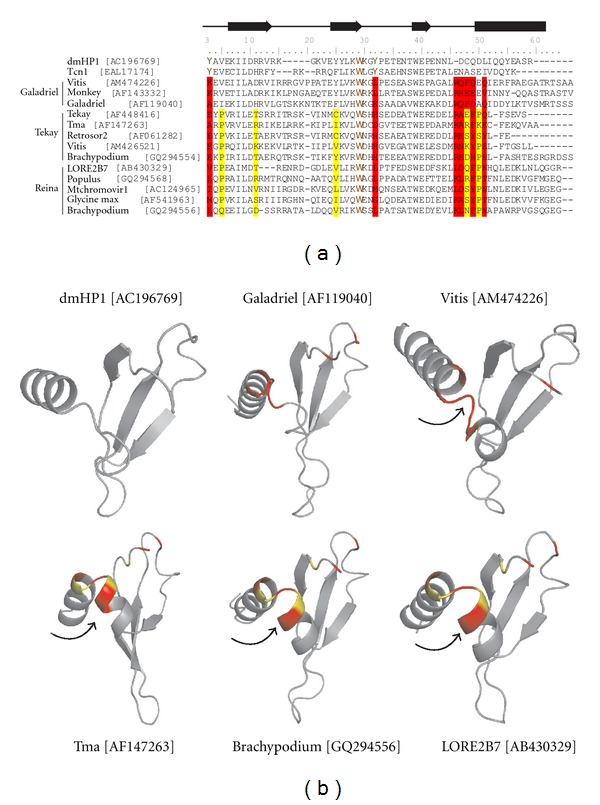
Multiple alignment including the “classical” chromodomain (dmHP1 from *Drosophila melanogaster*) and sampled representatives of group I and group II chromodomains from LTR retrotransposons (a); estimated tertiary structure for dmHP1 CHD (pdb : 1q3l; [7]) and predicted tertiary structures for some LTR retrotransposon CHDs (b). The GenBank accession numbers are indicated for each sequence used. The secondary structure elements are shownon the top (arrows indicate *β*-strands; rectangle *α*-helix) [3–7]. Three aminoacids that were shown to be under positive selection in the Tekay-Reina cluster are highlighted in yellow. The aminoacids that are potentially under positive selection in the Galadiriel-Reina-Tekay cluster are highlighted in red. An additional helix structure is indicated by an arrow for representatives of the Reina and Tekay clades.

The branch-site tests also revealed several events of positive selection on the CHD phylogenetic tree. The evolutionary changes of CHDs in the Galadriel-Tekay-Reina group most probably occurred under positive selection at the 0.05 significance level based on the mixture distribution by Hommel correction procedure (GTR branch on Figure 4(c)). Nonsynonymous substitutions were also significantly elevated above background on branches subtending the Tekay-Reina group and the Galadriel clade (branch site significance level of 0.1; TR and G branches on Figure 4(c), resp.). We found evidence for the positive selection of CHDs for the GTR and TR branches, with positive signals coming from a few codons (significance level 0.1; Figure 5). Specifically, three codons appeared to be under positive selection for the TR branch at the cutoff posterior probability 95% (corresponding to aminoacid resides in positions 3, 46, and 48 of the multiple alignments presented in Figure 5(a)). Proline residues that are located in positions 3 and 48 of CHDs from Tekay and Reina LTR retrotransposons are highly conserved in these clades (see also Figure 3). Such a high degree of conservation may indicate that these residues are functionally important for both the Reina- and Tekay-like CHDs. P3 is located in a position that corresponds to the residue V3 in “classical” CHDs and is believed to participate in the formation of a complementary surface that is responsible for histone 3 peptide (H3) recognition (based on a study of CHDs from histone protein 1, dmHP1, from *Drosophila melanogaster*; [3, 31–33].

The P48 and E46 residues are located in the area that corresponds to the helical structure in dmHP1 CHD and other CHDs. The presence of P48 in the region, which is expected to form an *α*-helix, should have significant effects on secondary structure. Prolines are rarely found in *α* and *β* structures, because the structure's side chain *α*-N can form only one hydrogen bond [37], which would reduce the stability of such structures. At the same time, prolines are easily accommodated in a variety of turns; for example, as a Pro-X corner (where X is a variable aminoacid residue) [38]. We reconstructed the tertiary structure of some representatives of plant retrotransposon CHDs using I-TASSER [39]. All of the representatives clearly exhibited the presence of tertiary structure similar to that of dmHP1 (Figure 5(b)). However, as expected due to the presence of P48, CHDs from LTR retrotransposons that belong to the Reina and Tekay clades bear additional helix structures in comparison to dmHP1. The P48 is located between the two helices and appears to be crucial for the helix-helix structure formation that is specific to plant LTR retrotransposon CHDs.

## 3. Discussion

The evolutionary history of retrotransposons includes the gain (and loss) of functional enzymatic domains, which allows them to adapt to a constantly changing genomic environment [12, 43–45]. The chromodomain (CHD) is believed to be a comparatively recent acquisition of LTR retrotransposons [12, 45]. The role of the chromodomains most likely is in targeting the insertion of new LTR retrotransposon copies into heterochromatic regions by recognizing specific heterochromatic histone marks and/or other factors [15]. As a consequence, LTR retrotransposons can easily avoid subsequent inactivation and elimination (purifying selection) because the chance to interfere with any coding sequence is small in heterochromatic regions [46].

**Figure 6:**
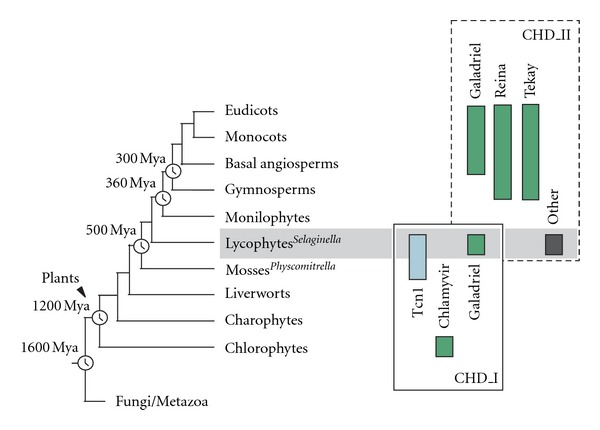
Distribution of different clades of chromodomain-containing LTR retrotransposons in plants as well as CHD_I and CHD_II. The evolutionary tree is represented according to Bowman et al., 2007 [40] and Berbee and Taylor, 2001 [41] with minor modifications. Divergence times (Mya: million years ago) are indicated according to Hedges, 2002 [42]. Other: SM-Diluvium and SM-Cranky.

Comparative and phylogenetic analysis demonstrates that plant LTR retrotransposon CHDs represent heterogeneous groups of enzymatic domains with a complex evolutionary history. First, it appears that plant retrotransposon CHD_II evolved from CHD_I, which can still be found in genomic sequences of green algae (Chlamyvir clade) and nonseed plants such as mosses and lycophytes (Tcn1 clade; Figure 6; and [13, 16, 18]). In addition, the SM1-Galahad and SM2-Galahad found in lycophyte *Selaginella* carried CHD_I, while belonged to the Galadriel clade of plant LTR retrotransposons. All of the other known representatives of this clade possess typical plant CHD_II domain. Lycophyte *Selaginella* appears to be a unique model species for the investigation of chromodomains among plants (Figure 6). This species is the only plant species known to have both types of retrotransposon CHDs in its genome. Moreover, LTR retrotransposon CHDs found in *Selaginella* represent transitional stages between CHD_I (found in fungi and animals) and CHD_II (described from angiosperms). Interestingly, the few CHDs that were found in gymnosperms are also distant from “typical” angiosperm CHD_II domain. We believe that the evolutionary history of plant LTR retrotransposon CHDs and their diversity among different plant groups reflect changes that occurred in chromatin organization (e.g., the distribution of heterochromatin/euchromatin mark; or molecular differences in specific heterochromatin/euchromatin marks) from green algae to higher plants. It was proposed earlier that although the histone methylation marks are conserved among eukaryotes, the distribution of the individual marks and their functional meaning may have diverged as different phyla evolved [47].

The occurrence of heterochromatic marks in plants differs from that of fungi and mammals. For example, histone H3 trimethyl-K9 (H3K9me3) is a heterochromatin-specific mark in *Schizosaccharomyces pombe *[48] and in mammals [49, 50] but has never been found to be associated with heterochromatin in plants (reviewed in [51]). Moreover, heterochromatin-specific marks have an uneven distribution among plants: histone H3 dimethyl-K27 (H3K27me2) has been shown to be a typical modification in the heterochromatic regions of *Arabidopsis thaliana*, *Vicea faba*, *Zea mays,* and *Secale cereale*, but it was not detected in species such as *Glycine max, Plantago ovate, *and *Hordeum vulgare *[52–58]; only two species so far, *S. cereale* and in *V. faba*, showed labeling of heterochromatin with histone H3 trimethyl-K27 (H3K27me3) [53, 55]. Very little is known about chromatin organization in mosses, lycophytes, and ferns [59]. The limited information that is available for gymnosperms indicates that their heterochromatic marks seem to be quite different from those of angiosperms [47]. For example, histone H3 monomethyl-K9 (H3K9me1), H3K9me2, and histone H3 monomethyl-K27 (H3K27me1) modifications, which are believed to be associated with silencing and heterochromatin formation in *A. thaliana*, are underrepresented in *Picea abies* and *Pinus sylvestris*. At the same time, H3K9me3 and H3K27me3 are typical heterochromatic marks in two gymnosperm species but are not present in *Arabidopsis *[47].

One would expect mobile elements to be very sensitive to any changes in host genome function and organization; they must adapt or be eliminated from the genome. Divergence in the distribution of individual heterochromatin-associated histone methylation marks could trigger the evolutionary changes of LTR retrotransposon CHDs in plants, which would result in a shift from the original CHD_I (still present in green algae, mosses, and lycophytes) to CHD_II (all higher plants) and subsequent subdivision into clade-specific CHDs (Tekay-, Reina-, and Galadriel-like CHDs). The initial plasticity of CHDs provided a wide range of possibilities for evolution. Chromodomains carry diverse functions in cells, from the recognition of specific H3 histone modifications to protein dimerisation as well as DNA and RNA binding (for review [3]). It is believed that considerable diversity of recognition by CHDs is generated within the CHD family through relatively few aminoacid substitutions at the aromatic cage or the peptide-binding sites [31]. While the function of retrotransposon CHDs is generally unknown, it was demonstrated that CHD_I of MAGGY LTR retrotransposon from rice-blast fungus *Magnaporthe oryzae *targets the integration of new copies to heterochromatin by recognizing H3K9me2 and H3K9me3 modifications [15]. Although a colocalization of TFL2, a dmHP1-like homolog in* Arabidopsis*, and CHD_II of the Tma LTR retrotransposon from *A. thaliana *were shown in the same study, the actual interacting factor(s) for plant CHD_II was not found.

The sequence divergence between CHD_I and CHD_II is a key to the functional differences, and positive selection appears to be involved in the diversification of CHDs during the evolutionary history of LTR retrotransposons. The presence of positive selection is uncommon among LTR retrotransposon domains [34–36]. It was proposed earlier that LTR retrotransposons rarely undergo substitution events that are driven by positive selection, which allows elements to remain unrecognized by the host genome and to escape silencing [36]. However, the CHD itself provides the possibility of escaping silencing by the specific targeting of heterochromatic regions when LTR retrotransposons integrate [15], and their rapid evolution could be advantageous. Most of the genes for which positive selection has been documented are involved in interactions between the organism and the environment (e.g., [60]) and/or are subjected to genetic conflict (e.g., [61–63]). What could be driving the evolution of chromodomains? One of the most attractive explanations is coevolutionary pressures; for example, plant LTR retrotransposon CHDs might evolve after heterochromatin-associated histone methylation marks the host species. This scenario could explain the shift from CHD_I to CHD_II after the divergence of the plant and fungi/metazoa groups as well as the divergence of CHDs between angiosperms and gymnosperms. It is possible that rapid adaptation coupled with subsequent strong selective pressure not only led to the adaptation of LTR retrotransposons to a changing “chromatin” environment in plants in general, but it also may have contributed to a functional diversification of clade-specific LTR retrotransposon CHDs. In other words, while Galadriel-, Reina- and Tekay-like CHDs were still involved in targeted-integration of new LTR retrotransposon copies, they could possibly recognize different chromatin marks or factors.

Mobile elements in general, and LTR retrotransposons in particular, are important parts of plant genomes. While it is still believed that mobile elements are selected against and silenced in host genomes to prevent their harmful effects, increasing numbers of studies indicate that mobile elements have been positively selected as major components of heterochromatin (see [64]). Chromodomain-containing LTR retrotransposons are the most remarkable example of mobile elements that developed the mechanism of targeted integration into heterochromatic regions. In the present study, we inferred that interactions between chromodomain-containing LTR retrotransposons and the host genome resulted in the present diversity of plant LTR retrotransposon CHDs and, most likely, led to the retrotransposon-enriched genome organization in plants. It is necessary to note that chromodomain-containing LTR retrotransposons in plant genomes represent a large pool of diverse chromatin remodeling domains, which also possess high evolutionary plasticity (for a review, see [3]). The potential roles of LTR retrotransposon chromodomains in genome and chromatin organization are poorly understood but should not be underestimated.

## 4. Materials and Methods

### 4.1. Genomic Sequence Screening and Sequence and Phylogenetic Analysis

*Selaginella moellendorffii *genomic sequence is available at the DOE Joint Genome Institute ([65]; http://genome.jgi-psf.org/Selmo1/Selmo1.info.html). We performed BLASTN and TBLASTN searches of the *S. moellendorffii* database (http://genome.jgi-psf.org/Selmo1/Selmo1.info.html; with default parameters) using the SM-Tcn1 retrotransposon [18] and previously described retrotransposons from other species as queries: Osr35—AC068924; rn377-208—AK068625; Reina—U69258; RIRE3—AC119148; Tekay—AF050455; Retrosor2—AF061282; Tma—AF147263; Galadriel—AF119040. The full-length copies of newly identified Gypsy LTR retrotransposons were discovered in genomic sequences and analyzed by UniPro uGENE software (http://ugene.unipro.ru/). The LTR retrotransposon sequences obtained during BLASTN and TBLASTN searches were localized using UniPro uGENE “Find pattern” option with default parameters (both strands, match percent: 100%, whole sequence range). Open reading frames were detected by UniPro uGENE “Find ORFs” option with default parameters. Pseudo-ORFs were manually reconstructed. Putative consensus sequences were reconstructed based on multiple alignments of copies. All DNA alignments were performed by ClustalW [66] with default parameters and were edited manually in UniPro uGENE.

The LTR retrotransposon chromodomain search was carried out using BLAST (BLASTN, TBLASTN, and BLASTP). BLAST analysis was performed using sequence databases that were accessible from the National Center for Biotechnology Information (NCBI) server (http://blast.ncbi.nlm.nih.gov/Blast.cgi; BLASTP and MegaBLAST with default parameters), the U.S. Department of Energy Joint Genome Institute (http://genome.jgi-psf.org/), PlantGDB (http://www.plantgdb.org/; BLASTN, TBLASTN and BLASTP with default parameters), and Phytozome, a tool for green plant comparative genomics (http://www.phytozome.net/; BLASTN with default parameters). The full list of species investigated and primary information can be found in Supporting Information Table 1S and Table 2S. Aminoacid sequences of the known CHDs were used as queries: MAGGY—L35053; Tcn1—XM_571377; Retrosor2—AF061282; Tma—AF147263; Galadriel—AF119040. To discriminate between functional proteins and retrotransposon CHDs, the next round of BLASTP was performed using newly identified CHDs as queries.

All multiple alignments were performed by ClustalW [66] and were edited manually in UniPro uGENE (http://ugene.unipro.ru/). Phylogenetic analyses were performed using the Maximum Likelihood (ML) method in the PhyML 3.0 program [67]. Neighbor-Joining analysis was performed using the MEGA5 software [68]. Statistical support for the NJ tree was evaluated by bootstrapping (number of replications, 1000) [69]. Statistical support for the ML tree was evaluated by approximate likelihood ratio test (aLRT; Figure 2) and by bootstrapping (number of replications, 100; Figure 4) [69, 70]. The tertiary structures of investigated CHD peptides were predicted using I-TASSER server with default parameters (http://zhanglab.ccmb.med.umich.edu/I-TASSER/) [39]. The tertiary structure of dmHP1 from *Drosophila melanogaster* is available in Protein Data Bank (http://www.rcsb.org/pdb/home/home.do) under the ID—1q3l [7].

### 4.2. Test for Selection

The multiple alignment of CHD sequences was performed using ClustalW [66] available at the RevTrans 1.4 Server ([71]; http://www.cbs.dtu.dk/services/RevTrans). RevTrans takes a set of DNA sequences, virtually translates them, aligns the peptide sequences, and uses this as a scaffold for constructing the corresponding DNA multiple alignment. The phylogenetic trees of domains (Figure 4) were obtained with the maximum likelihood algorithm implemented in the PhyML 3.0 program [67]. From nucleotide sequence alignments for each domain, we reconstructed the phylogenetic trees under HKY85 + G model. The PhyML tree searching algorithm was chosen as the best of subtree pruning and regrafting (SPR) and nearest neighbor interchange (NNI) for more thorough explorations of the space of topologies. To assess the reliability of the reconstructed phylogenies, we performed 100 bootstrap reconstructions for each domain [69].

The nonsynonymous to synonymous substitution rate ratio (*ω* = *d*_*N*_/*d*_*S*_) provides a measure of natural selection at the protein level, with *ω* = 1, >1, and <1, indicating neutral evolution, purifying selection, and positive selection, respectively. We use codeml program (the PAML package) to perform lineage and clade-specific analyses of *d*_*N*_/*d*_*S*_ ratios (*ω*) [72–74]. The program codeml implements large collection of codon substitution models. Positive selection is tested using a likelihood ratio test (LRT) comparing a null model that does not allow *ω* > 1 with an alternative model that does. When two models are nested, twice the log-likelihood difference between the two models can be compared with the *χ* ^2^ distribution, with the difference in the number of parameters between the two models as the degrees of freedom (df).

At first, to evaluate whether any of three chosen domains from four diverse clades are undergoing positive selection affecting all sites over prolonged time, the simplest one-ratio site model (M0) was used (codeml parameters used were as follows: model = 0, NSsites = 0). One should keep in mind, since our null hypothesis was always the absence of positive selection failing to reject a null hypothesis and/or providing a null hypothesis were interpreted as an absence of positive selection in either case. At the second stage, we tested each of the clades/groups for a signature of positive selection using clade-site test, this compares the modified clade model C (model = 3, NSsites = 2) with the neutral M1a model (model = 0, NSsites = 0). For this comparison df was set to 3. We conducted one specific test for each domain using extension of clade model C which allows for more than two branch types. Branches leading to appropriate clade/group were labeled: Tekay: T; Reina: R; Galadriel: G; Tekay-Reina: TR; Galadriel-Tekay-Reina: GTR; Reina-Galadriel: RG; Tekay-Reina-Galadriel: TRG, other branches were used as “background”. The main purpose of the test was to identify whether there is at least one branch potentially under positive selection. All maximum likelihood estimates for RT branches of the site class 2 *ω* ratio were close to 0, thus taking in the consideration previous results we conclude that there are not any events of positive selection. For CHD and Int domains there was at least one estimate greater than one. Nevertheless, this clade-based test does not directly examine whether any *ω* ratio is significantly greater than one.

Finally, we applied the LRT based on two branch-site models with one, where *ω* was to be estimated and other with *ω* fixed to 1, to test every of five aforementioned branch of the CHD and Int trees for evidence of positive selection, this test is also known as the branch-site test of positive selection. Branch-site models of codon substitution allow *ω* to vary both among sites in the protein and across branches on the tree and provide a means to detect short episodes of molecular adaptation affecting just a few sites [75]. In these models it is assumed that the branches are *a priori* divided into foreground and background. Only foreground lineages may have experienced positive selection. One of two branch-site models presented in codeml—modified model A (model = 2, NSsites = 2) was used for comparison with null hypothesis [75]. The model assumes four classes of sites. Site class 0 includes codons that are conserved throughout the tree, with 0 < *ω*_0_ < 1 estimated. Site class 1 includes codons that are evolving neutrally throughout the tree with *ω*_1_ = 1. Site classes 2a and 2b include codons that are conserved or neutral on the background branches, but become under positive selection on the foreground branches with *ω*_2_ > 1 estimated. The model involves four parameters in the *ω* distribution: *p*_0_, *p*_1_, *ω*_0_, and *ω*_2_. The null hypothesis was also modified model A but with *ω*_2_ = 1 fixed (codeml options were switched from fix_omega = 0 to fix_omega = 1 and omega = 1). A likelihood ratio test (LRT) based on models was found to have satisfactory accuracy and reasonable power [75–79]. Branch-site models allow only two types of branches thus the most common approach to test several branches on the tree is to treat every branch as foreground in turn. The probability of rejecting falsely at least one of null hypotheses in such tests can be high. The correction for multiple testing becomes necessary. The Hommel procedure that controls family-wise error rate (FWER) was used as correction method. The results of Bayes empirical Bayes (BEB) approach which accommodates uncertainties in the maximum likelihood estimates were used to identify sites under positive selection if the likelihood ratio test after correction procedure was significant. Table 3 summarizes maximum likelihood estimates and test statistics for LRTs corresponding to the every of 5 branches leading to appropriate clades/groups of the CHD tree (see Supporting Information Protocol S1 for details).

## Supplementary Material

Supplementary material Table S1 contains list of Plant species used in this study, their taxonomy (according to NCBI Taxonomy: http://www.ncbi.nlm.nih.gov/taxonomy) and results of in silico mining of chromodomains by LTR retrotransposons clades.
Supplementary material Table S2 contains list of species genomes of which were analyzed *in silico* in present study and the sources of the ESTs and/or genomic sequences.
Supplementary material Figure 1S represents the detailed Maximum likelihood (ML) phylogenetic tree (showed in Figure 2) based on LTR retrotransposon chromodomain (CHD) aminoacid sequences, including CHDs from newly identified chromodomain-containing LTR retrotransposons from *Selaginella moellendorffii*. Statistical support was evaluated by using aLTR; nodes with aLRT statistics over 50% are shown. The plant-specific clades, Chlamyvir, Reina, CRM, Galadriel, and Tekay, as well as the Tcn1 clade, are indicated. The name of the host species and the accession number is indicated for some of the LTR elements taken from GenBank. The taxonomic range of host species is indicated by colored boxes and includes angiosperms, gymnosperms, green algae, and non-seed plants (*Selaginella* and *Physcomitrella*).
Supplementary material Protocol S1 provides detailed positive selection test results.

## Figures and Tables

**Table 1:** The characteristics of chromodomain-containing Gypsy LTR retrotransposons from *Selaginella moellendorffii*.

Element name	Element size [bp]	Intact copies	LTRs size [bp]	LTRs identity [%]	TSD	ITR
SM1-Galahad*	~7500	ND	1076/1146	90.2	ND	TG*⋯*CA
SM2-Galahad	7353	+	919	99.7	CCTAT*⋯*CCTAT	TGT*⋯*ACA
SM-Fogey	4916	+	233	98.7	TGCCC*⋯*TGCCC	TG*⋯*CA
SM-Diluvium	5038	+	112	97.3	GCGTA*⋯*GCGTA	TG*⋯*CA
SM-Cranky	5594	—	217	98.6	GTTTCT*⋯*GTTTCT	TG*⋯*CA

*SM1-Galahad was reconstructed based on a number of sequences; TSD: terminal site duplications; ITR: dinucleotide inverted repeat; ND: not detected.

**Table 2:** List of some plant species used in this study, their taxonomy (according to NCBI Taxonomy: http://www.ncbi.nlm.nih.gov/taxonomy) and the results of *in silico* mining of chromodomains by LTR retrotransposon clades. The full list is available in Supporting Information Table S1 in Supplementary Material available online at doi: 10.1155/2012/874743.

Class	Order	Family	Species		EST		GSS/WGS		
				Chlamyvir	Other		Chlamyvir	other	

Bangiophyceae	Bangiales	Bangiaceae	*Porphyra yezoensis*	—	—		NA	NA	

	Cyanidiales	Cyanidiaceae	*Galdieria sulphuraria*	—	—		NA	NA	

Trebouxiophyceae	Chlorellales	Chlorellaceae	*Chlorella vulgaris*	—	—		7	—	

Chlorophyceae	Chlamydomonadales	Chlamydomonadaceae	*Chlamydomonas reinhardtii*	—	—		13	3	

		Volvocaceae	*Volvox carteri f. nagariensis*	3	—		241	20	

				Reina	Tekay	Galadriel	Reina	Tekay	Galadriel

Lycopodiopsida	Lycopodiales	Lycopodiaceae	*Huperzia serrata*	—	—	—	NA	NA	NA

Polypodiopsida	Polypodiales	Pteridaceae	*Adiantum capillus-veneris*	—	1	—	NA	NA	NA

Coniferopsida	Coniferales	Pinaceae	*Picea glauca *	6	2	—	—	—	—

			*Pinus taeda*	4	5	—	18	2	—

			*Pinus banksiana*	9	3	—	—	—	—

Monocotyledons	Poales	Poaceae	*Triticum aestivum *	37	5	—	10	91	—

			*Brachypodium distachyon*	11	—	—	46	371	—

			*Oryza sativa Indica Group*	5	1	—	64	239	—

			*Oryza sativa Japonica Group*	55	6	—	23	78	—

			*Sorghum bicolor*	14	—	—	18	>1000	—

			*Zea mays*	53	105	—	600	>10000	—

Eudicotyledons	Solanales	Solanaceae	*Solanum lycopersicum*	—	11	—	17	>1000	80

			*Solanum tuberosum*	2	8	1	11	>1000	12

			*Nicotiana tabacum*	23	39	2	185	2973	297

	Lamiales	Phrymaceae	*Mimulus guttatus*	2	—	1	16	370	13

	Brassicales	Brassicaceae	*Arabidopsis thaliana*	2	6	—	14	23	—

			*Brassica napus*	2	3	—	5	99	2

	Sapindales	Rutaceae	*Citrus clementina*	—	—	1	11	8	35

	Fabales	Fabaceae	*Glycine max*	1	2	—	22	252	—

			*Lotus japonicus*	2	3	—	8	62	—

	Vitales	Vitaceae	*Vitis vinifera*	—	4	4	18	59	327

NA: no data available.

**Table 3:** Maximum likelihood estimates and LRT statistics for the chromodomain.

Foreground branch	2Δ*ℓ*	*p*_1/2*χ*_0_^2^+1/2*χ*_1_^2^_	${\hat{p}}_{0}$	${\hat{p}}_{1}$	${\hat{\omega }}_{0}$	${\hat{\omega }}_{2}$	Positively selected sites (90%)
base T	1.634722	0.1005255	0.71854	0.02675	0.07183	∞	
base R	1.11799	0.145176	0.69307	0.02571	0.07074	19.50879	
base G*	**4.766098**	**0.014513**	**0.84189**	**0.03127**	**0.07093**	**∞**	none
base TR*	**3.720944**	**0.026867**	**0.70023**	**0.02601**	**0.07199**	**∞**	3/9/23/46/48
base GTR*	**5.652052**	**0.0087175**	**0.71684**	**0.02660**	**0.07097**	**∞**	1/30/44/45/46/47/49

*Branches Galadriel (G), Tekay-Reina (TR), Galadriel-Tekay-Reina (GTR) are detected to be under positive selection by Hommel test procedure.
