# Supplementary material for: Evolutionary History of LTR Retrotransposon Chromodomains in Plants
Source: Int J Plant Genomics. 2012 Apr 29;2012:874743. doi: 10.1155/2012/874743 (PMC3350952; doi:10.1155/2012/874743)
Supplement: Supplementary file 1 — Supplementary material Table S1 contains list of Plant species used in this study, their taxonomy (according to NCBI Taxonomy: http://www.ncbi.nlm.nih.gov/taxonomy) and results of in silico mining of chromodomains by LTR retrotransposons clades. Supplementary material Table S2 contains list of species genomes of which were analyzed in silico in present study and the sources of the ESTs and/or genomic sequences. Supplementary material Figure 1S represents the detailed Maximum likelihood (ML) phylogenetic tree (showed in Figure 2) based on LTR retrotransposon chromodomain (CHD) aminoacid sequences, including CHDs from newly identified chromodomain-containing LTR retrotransposons from Selaginella moellendorffii. Statistical support was evaluated by using aLTR; nodes with aLRT statistics over 50% are shown. The plant-specific clades, Chlamyvir, Reina, CRM, Galadriel, and Tekay, as well as the Tcn1 clade, are indicated. The name of the host species and the accession number is indicated for some of the LTR elements taken from GenBank. The taxonomic range of host species is indicated by colored boxes and includes angiosperms, gymnosperms, green algae, and non-seed plants (Selaginella and Physcomitrella). Supplementary material Protocol S1 provides detailed positive selection test results. [file 874743.f1.pdf]

Supplementary material Table S1. List of Plant species used in this study, their taxonomy (according to NCBI Taxonomy: http://www.ncbi.nlm.nih.gov/taxonomy) and results of *in silico* mining of chromodomains by LTR retrotransposons clades.

Class	Order	Family	Tribe	Species	EST			GSS/WGS		
					Chlamyvir	other		Chlamyvir	other	
Bangiophyceae	Bangiales	Bangiaceae		*Porphyra yezoensis*	--	--		ND	ND	
	Cyanidiales	Cyanidiaceae		*Galdieria sulphuraria*	--	--		ND	ND	
				Cyanidioschyzon merolae	ND	ND		--	--	
Trebouxiophyceae	Chlorellales	Chlorellaceae		*Chlorella vulgaris*	--	--		7	--	
Chlorophyceae	Chlamydomonadales	Chlamydomonadaceae		*Chlamydomonas reinhardtii*	--	--		13	3	
				Volvox carteri f nagariensis	3	--		241	20	
Prasinophyceae		Mamiellaceae		*Micromonas pusilla*	ND	ND		--	--	
				Ostreococcus lucimarinus	ND	ND		--	--	
				Ostreococcus tauri	ND	ND		--	--	
					Reina	Tekay	Galadriel	Reina	Tekay	Galadriel
Lycopodiopsida	Lycopodiales	Lycopodiaceae		*Huperzia serrata*	--	--	--	ND	ND	ND
Polypodiopsida	Polypodiales	Pteridaceae		*Adiantum capillus-veneris*	--	1	--	ND	ND	ND
Coniferopsida	Coniferales	Pinaceae		*Picea glauca*	6	2	--	--	--	--
				Picea sitchensis	5	5	--	ND	ND	ND
				Pinus taeda	4	5	--	18	2	--
				Pinus banksiana	9	3	--	--	--	--
				Pinus contorta	2	3	--	ND	ND	ND
				Pseudotsuga menziesii var. menziesii	2	1	--	ND	ND	ND
				Pseudotsuga menziesii	1	--	--	ND	ND	ND
				Cryptomeria japonica	2	--	--	ND	ND	ND
Monocotyledons	Alismatales	Araceae		*Wolffia arrhiza*	--	--	--	ND	ND	ND
	Poales	Poaceae	Poeae	*Agrostis stolonifera*	1	6	--	ND	ND	ND
				Festuca arundinacea	19	4	--	1	10	--
				Festuca pratensis	20	7	--	ND	ND	ND
				Puccinellia tenuiflora	--	--	--	ND	ND	ND
			Triticeae	*Hordeum vulgare*	--	2	--	--	3	--
				Hordeum vulgare subsp. vulgare	5	5	--	--	--	--
				Leymus cinereus x Leymus triticoides	1	--	--	ND	ND	ND
				Triticum aestivum	37	5	--	10	91	--
				Triticum monococcum	--	--	--	--	--	--
				Triticum turgidum subsp durum	2	2	--	--	--	--
				Pseudoroegneria spicata	--	--	--	ND	ND	ND
			Brachypodieae	*Brachypodium distachyon*	11	--	--	46	371	--
			Oryzeae	*Oryza sativa*	2	1	--	--	8	--
				Oryza sativa Indica Group	5	1	--	64	239	--
				Oryza sativa Japonica Group	55	6	--	23	78	--
			Paniceae	*Panicum virgatum*	11	1	--	ND	ND	ND
				Pennisetum glaucum	--	1	--	--	--	--
			Andropogoneae	*Saccharum hybrid cultivar CP72 2086*	1	--	--	ND	ND	ND
				Saccharum officinarum	12	6	--	ND	ND	ND
				Sorghum bicolor	14	--	--	18	>1000	--
				Zea mays	53	105	--	600	>10000	--
			Cynodonteae	*Cynodon dactylon*	--	--	--	--	--	--

Supplementary material Table S1 (continued). List of Plant species used in this study, their taxonomy (according to NCBI Taxonomy: http://www.ncbi.nlm.nih.gov/taxonomy) and results of *in silico* mining of chromodomains by LTR retrotransposons clades.

	Order	Family	Tribe	Species	EST			GSS/WGS		
					Reina	Tekay	Galadriel	Reina	Tekay	Galadriel
Monocotyledons	Poales	Poaceae	Eragrostideae	*Eragrostis curvula*	--	--	--	ND	ND	ND
	Zingiberales	Zingiberaceae		*Zingiber officinale*	--	--	--	ND	ND	ND
Eudicotyledons	Caryophyllales	Amaranthaceae		*Beta vulgaris*	--	--	1	7	39	--
		Aizoaceae		*Mesembryanthemum crystallinum*	--	--	--	ND	ND	ND
		Tamaricaceae		*Tamarix hispida*	--	--	--	ND	ND	ND
	Ericales	Actinidaceae		*Actinidia chinensis*	--	1	--	ND	ND	ND
				Actinidia deliciosa	2	9	--	--	--	--
		Theaceae		*Camellia sinensis*	--	--	--	--	--	--
	Asterales	Asteraceae	Anthemideae	*Artemisia annua*	--	14	--	--	--	--
			Heliantheae	*Helianthus annuus*	5	2	3	1	4	--
				Zinnia violacea	--	3	--	ND	ND	ND
			Mutisieae	*Gerbera hybrid cultivar*	--	--	1	ND	ND	ND
			Cichorieae	*Lactuca sativa*	--	1	--	--	--	--
				Lactuca serriola	--	--	1	ND	ND	ND
	Solanales	Solanaceae	Capsiceae	*Capsicum annuum*	11	4	--	--	--	--
			Solaneae	*Solanum habrochaites*	--	2	--	ND	ND	ND
				Solanum lycopersicum	--	11	--	17	>1000	80
				Solanum melongena	--	4	--	--	1	--
				Solanum torvum	--	3	3	ND	ND	ND
				Solanum tuberosum	2	8	1	11	>1000	12
			Nicotianeae	*Nicotiana benthamiana*	14	--	--	18	>1000	--
				Nicotiana tabacum	23	39	2	185	2973	297
	Lamiales	Phrymaceae		*Mimulus guttatus*	2	--	1	16	370	13
				Mimulus lewisii	--	--	--	4	--	--
		Orobanchaceae	Buchnereae	*Striga hermonthica*	--	2	--	10	34	1
			Rhinantheae	*Triphysaria versicolor*	--	--	--	ND	ND	ND
	Gentianales	Apocynaceae	Vinceae	*Catharanthus roseus*	--	--	--	1	--	--
		Rubiaceae	Coffeeae	*Coffea arabica*	--	--	--	1	6	--
	Apiales	Araliaceae		*Eleutherococcus sessiliflorus*	--	--	--	ND	ND	ND
				Panax ginseng	--	--	--	--	5	--
	Brassicales	Brassicaceae		*Arabidopsis thaliana*	2	6	--	14	23	--
				Brassica napus	2	3	--	5	99	2
				Brassica oleracea	--	--	--	53	101	90
				Brassica rapa	--	--	2	2	7	2
				Raphanus sativus	--	1	--	--	--	--
				Raphanus raphanistrum subsp. raphanistrum	--	--	--	ND	ND	ND
				Tropaeolum majus	--	--	--	ND	ND	ND
	Sapindales	Rutaceae		*Citrus clementina*	--	--	1	11	8	35
				Citrus sinensis	2	5	3	ND	ND	ND
				Citrus trifoliata	--	1	--	ND	ND	ND
				Citrus unshiu	--	--	1	ND	ND	ND

Supplementary material Table S1 (continued). List of Plant species used in this study, their taxonomy (according to NCBI Taxonomy: http://www.ncbi.nlm.nih.gov/taxonomy) and results of *in silico* mining of chromodomains by LTR retrotransposons clades.

	Order	Family	Tribe	Species	EST			GSS/WGS		
					Reina	Tekay	Galadriel	Reina	Tekay	Galadriel
Eudicotyledons	Malvales	Malvaceae		*Gossypium arboreum*	--	6	--	--	--	--
				Gossypium hirsutum	3	17	3	3	406	17
				Gossypium raimondii	1	2	--	1	91	4
				Theobroma cacao	--	3	--	--	--	--
	Myrtales	Myrtaceae		*Eucalyptus gunnii*	--	--	--	ND	ND	ND
	Fabales	Fabaceae	Dalbergieae	*Arachis hypogaea*	3	3	1	--	9	--
			Phaseoleae	*Glycine max*	1	2	--	22	252	--
				Glycine soja	--	--	--	17	306	--
				Vigna unguiculata	3	--	--	7	99	--
				Cajanus cajan	--	--	--	11	113	--
			Loteae	*Lotus japonicus*	2	3	--	8	62	--
			Trifolieae	*Medicago sativa*	1	1	--	--	--	--
				Medicago truncatula	--	2	--	3	322	--
				Trifolium pratense	--	--	--	1	8	--
			Phaseoleae	*Phaseolus vulgaris*	--	2	--	--	--	--
				Phaseolus angustissimus	--	--	--	ND	ND	ND
			Fabeae	*Pisum sativum*	1	--	--	--	--	--
			Cicereae	*Cicer arietinum*	--	--	--	12	309	--
			Galegeae	*Glycyrrhiza uralensis*	--	--	--	ND	ND	ND
	Cucurbitales	Cucurbitaceae		*Cucumis sativus*	--	--	1	1	2	--
	Rosales	Rosaceae	Potentilleae	*Fragaria vesca*	--	--	4	ND	ND	ND
			Pyreae	*Malus x domestica*	3	1	--	--	--	--
			Amygdaleae	*Prunus persica*	--	1	--	1	18	4
		Cannabaceae		*Humulus lupulus*	1	--	--	ND	ND	ND
				Cannabis sativa	--	--	--	ND	ND	ND
	Fagales	Juglandaceae		*Juglans hindsii x Juglans regia*	1	--	--	ND	ND	ND
		Fagaceae		*Quercus robur*	1	--	--	ND	ND	ND
		Betulaceae		*Betula platyphylla*	--	--	--	ND	ND	ND
	Malpighiales	Euphorbiaceae	Manihoteae	*Manihot esculenta*	--	2	2	1	31	--
		Salicaceae	Saliceae	*Populus tremula x Populus tremuloides*	--	1	--	ND	ND	ND
				Populus trichocarpa x Populus deltoides	3	1	--	ND	ND	ND
	Vitales	Vitaceae		*Vitis vinifera*	--	4	4	18	59	327
	Ranunculales	Ranunculaceae		*Aquilegia formosa x Aquilegia pubescens*	5	--	--	ND	ND	ND
		Papaveraceae		*Papaver somniferum*	7	3	2	ND	ND	ND
	Malvales	Malvaceae		*Gossypium arboreum*	--	6	--	--	--	--
				Gossypium hirsutum	3	17	3	3	406	17
				Gossypium raimondii	1	2	--	1	91	4

Supplementary material Table S2. List of species genomes of which were analyzed *in silico* in present study and the sources of the ESTs and/or genomic sequences.

Class	Order	Family	Species	Organization: Web link
Bangiophyceae		Bangiaceae	*Porphyra yezoensis*	KDRI: http://est.kazusa.or.jp/en/plant/porphyra/EST/
		Cyanidiaceae	*Galdieria sulphuraria*	MSU: http://genomics.msu.edu/galdieria/
			Cyanidioschyzon merolae	http://merolae.biol.s.u-tokyo.ac.jp/
Trebouxiophyceae	Chlorellales	Chlorellaceae	*Chlorella vulgaris*	JGI: http://genome.jgi-psf.org/Chlvu1/Chlvu1.home.html
Chlorophyceae		Chlamydomonadaceae	*Chlamydomonas reinhardtii*	KDRI: http://est.kazusa.or.jp/en/plant/chlamy/EST/ PlantGDB: http://www.plantgdb.org/prj/ESTCluster/ JGI Phytozome: http://www.phytozome.net/
Prasinophyceae		Mamiellaceae	*Micromonas pusilla*	JGI: http://genome.jgi-psf.org/MicpuC1/MicpuC1.home.html
			Ostreococcus lucimarinus	PlantGDB: http://www.plantgdb.org/prj/ESTCluster/
			Ostreococcus tauri	JGI: http://genome.jgi-psf.org/Ostta4/Ostta4.home.html
			Volvox carteri f nagariensis	JGI Phytozome: http://www.phytozome.net/ JGI: http://genome.jgi-psf.org/Volca1/Volca1.home.html PlantGDB: http://www.plantgdb.org/prj/ESTCluster/
Lycopodiopsida	Lycopodiales	Lycopodiaceae	*Huperzia serrata*	NCBI SRA: http://www.ncbi.nlm.nih.gov/sra/SRX010999
Polypodiopsida	Polypodiales	Pteridaceae	*Adiantum capillus-veneris*	PlantGDB: http://www.plantgdb.org/prj/ESTCluster/
Coniferopsida	Coniferales	Pinaceae	*Picea glauca*	PlantGDB: http://www.plantgdb.org/prj/ESTCluster/
			Picea sitchensis	PlantGDB: http://www.plantgdb.org/prj/ESTCluster/
			Pinus taeda	MGEL: http://www.pine.msstate.edu/seq.htm PlantGDB: http://www.plantgdb.org/prj/ESTCluster/
			Pinus banksiana	PlantGDB: http://www.plantgdb.org/prj/ESTCluster/
			Pinus contorta	PlantGDB: http://www.plantgdb.org/prj/ESTCluster/
			Pseudotsuga menziesii var. menziesii	PlantGDB: http://www.plantgdb.org/prj/ESTCluster/
			Pseudotsuga menziesii	PlantGDB: http://www.plantgdb.org/prj/ESTCluster/
			Cryptomeria japonica	PlantGDB: http://www.plantgdb.org/prj/ESTCluster/
Monocotyledons	Alismatales	Araceae	*Wolffia arrhiza*	PlantGDB: http://www.plantgdb.org/prj/ESTCluster/
	Poales	Poaceae	*Agrostis stolonifera*	PlantGDB: http://www.plantgdb.org/prj/ESTCluster/
			Festuca arundinacea	PlantGDB: http://www.plantgdb.org/prj/ESTCluster/
			Festuca pratensis	PlantGDB: http://www.plantgdb.org/prj/ESTCluster/
			Hordeum vulgare	PlantGDB: http://www.plantgdb.org/HvGDB/
			Hordeum vulgare subsp. vulgare	PlantGDB: http://www.plantgdb.org/prj/ESTCluster/
			Leymus cinereus x Leymus triticoides	PlantGDB: http://www.plantgdb.org/prj/ESTCluster/
			Triticum aestivum	PlantGDB: http://www.plantgdb.org/prj/ESTCluster/
			Triticum monococcum	PlantGDB: http://www.plantgdb.org/prj/ESTCluster/
			Triticum turgidum subsp durum	PlantGDB: http://www.plantgdb.org/prj/ESTCluster/
			Pseudoroegneria spicata	PlantGDB: http://www.plantgdb.org/prj/ESTCluster/
			Brachypodium distachyon	JGI Phytozome: http://www.phytozome.net/ PlantGBD: http://www.plantgdb.org/BdGDB/ PlantGDB: http://www.plantgdb.org/prj/ESTCluster/
			Oryza sativa	JGI Phytozome: http://www.phytozome.net/ PlantGDB: http://www.plantgdb.org/prj/ESTCluster/
			Oryza sativa Indica Group	PlantGDB: http://www.plantgdb.org/prj/ESTCluster/
			Oryza sativa Japonica Group	PlantGDB: http://www.plantgdb.org/prj/ESTCluster/
			Panicum virgatum	PlantGDB: http://www.plantgdb.org/prj/ESTCluster/

Supplementary material Table S2 (continued). List of species genomes of which were analyzed *in silico* in present study and the sources of the ESTs and/or genomic sequences.

Class	Order	Family	Species	Organization: Web link
			Saccharum hybrid cultivar CP72 2086	PlantGDB: http://www.plantgdb.org/prj/ESTCluster/
			Saccharum officinarum	PlantGDB: http://www.plantgdb.org/prj/ESTCluster/
			Sorghum bicolor	JGI Phytozome: http://www.phytozome.net/<br>PlantGDB: http://www.plantgdb.org/prj/ESTCluster/
			Zea mays	JGI Phytozome: http://www.phytozome.net/<br>PlantGDB: http://www.plantgdb.org/prj/ESTCluster/
			Cynodon dactylon	PlantGDB: http://www.plantgdb.org/prj/ESTCluster/
			Eragrostis curvula	PlantGDB: http://www.plantgdb.org/prj/ESTCluster/
	Zingiberales	Zingiberaceae	*Zingiber officinale*	PlantGDB: http://www.plantgdb.org/prj/ESTCluster/
Eudicotyledons	Caryophyllales	Amaranthaceae	*Beta vulgaris*	PlantGDB: http://www.plantgdb.org/prj/ESTCluster/
		Aizoaceae	*Mesembryanthemum crystallinum*	PlantGDB: http://www.plantgdb.org/prj/ESTCluster/
		Tamaricaceae	*Tamarix hispida*	PlantGDB: http://www.plantgdb.org/prj/ESTCluster/
	Ericales	Actinidaceae	*Actinidia chinensis*	PlantGDB: http://www.plantgdb.org/prj/ESTCluster/
			Actinidia deliciosa	PlantGDB: http://www.plantgdb.org/prj/ESTCluster/
		Theaceae	*Camellia sinensis*	PlantGDB: http://www.plantgdb.org/prj/ESTCluster/
	Asterales	Asteraceae	*Artemisia annua*	PlantGDB: http://www.plantgdb.org/prj/ESTCluster/
			Helianthus annuus	PlantGDB: http://www.plantgdb.org/prj/ESTCluster/
			Zinnia violacea	PlantGDB: http://www.plantgdb.org/prj/ESTCluster/
			Gerbera hybrid cultivar	PlantGDB: http://www.plantgdb.org/prj/ESTCluster/
			Lactuca sativa	PlantGDB: http://www.plantgdb.org/prj/ESTCluster/
			Lactuca serriola	PlantGDB: http://www.plantgdb.org/prj/ESTCluster/
	Solanales	Solanaceae	*Capsicum annuum*	PlantGDB: http://www.plantgdb.org/prj/ESTCluster/
			Solanum habrochaites	PlantGDB: http://www.plantgdb.org/prj/ESTCluster/
			Solanum lycopersicum	PlantGDB: http://www.plantgdb.org/prj/ESTCluster/
			Solanum melongena	PlantGDB: http://www.plantgdb.org/prj/ESTCluster/
			Solanum torvum	PlantGDB: http://www.plantgdb.org/prj/ESTCluster/
			Solanum tuberosum	PlantGDB: http://www.plantgdb.org/prj/ESTCluster/
			Nicotiana benthamiana	PlantGDB: http://www.plantgdb.org/prj/ESTCluster/
			Nicotiana tabacum	PlantGDB: http://www.plantgdb.org/prj/ESTCluster/
	Lamiales	Phrymaceae	*Mimulus guttatus*	JGI Phytozome: http://www.phytozome.net/<br>PlantGDB: http://www.plantgdb.org/prj/ESTCluster/
			Mimulus lewisii	PlantGDB: http://www.plantgdb.org/prj/ESTCluster/
		Orobanchaceae	*Striga hermonthica*	PlantGDB: http://www.plantgdb.org/prj/ESTCluster/
			Triphysaria versicolor	PlantGDB: http://www.plantgdb.org/prj/ESTCluster/
	Gentianales	Apocynaceae	*Catharanthus roseus*	PlantGDB: http://www.plantgdb.org/prj/ESTCluster/
		Rubiaceae	*Coffea arabica*	PlantGDB: http://www.plantgdb.org/prj/ESTCluster/
	Apiales	Araliaceae	*Eleutherococcus sessiliflorus*	PlantGDB: http://www.plantgdb.org/prj/ESTCluster/
			Panax ginseng	PlantGDB: http://www.plantgdb.org/prj/ESTCluster/
	Brassicales	Brassicaceae	*Arabidopsis thaliana*	PlantGDB: http://www.plantgdb.org/prj/ESTCluster/
			Brassica napus	PlantGDB: http://www.plantgdb.org/prj/ESTCluster/
			Brassica oleracea	PlantGDB: http://www.plantgdb.org/prj/ESTCluster/
			Brassica rapa	PlantGDB: http://www.plantgdb.org/prj/ESTCluster/
			Raphanus sativus	PlantGDB: http://www.plantgdb.org/prj/ESTCluster/

Supplementary material Table S2 (continued). List of species genomes of which were analyzed *in silico* in present study and the sources of the ESTs and/or genomic sequences.

Class	Order	Family	Species	Organization: Web link
Eudicotyledons			*Raphanus raphanistrum subsp. raphanistrum*	PlantGDB: http://www.plantgdb.org/prj/ESTCluster/
			Tropaeolum majus	PlantGDB: http://www.plantgdb.org/prj/ESTCluster/
	Sapindales	Rutaceae	*Citrus clementina*	JGI Phytozome: http://www.phytozome.net/<br>PlantGDB: http://www.plantgdb.org/prj/ESTCluster/
			Citrus sinensis	JGI Phytozome: http://www.phytozome.net/<br>PlantGDB: http://www.plantgdb.org/prj/ESTCluster/
			Citrus trifoliata	PlantGDB: http://www.plantgdb.org/prj/ESTCluster/
			Citrus unshiu	PlantGDB: http://www.plantgdb.org/prj/ESTCluster/
	Myrtales	Myrtaceae	*Eucalyptus gunnii*	PlantGDB: http://www.plantgdb.org/prj/ESTCluster/
	Fabales	Fabaceae	*Arachis hypogaea*	PlantGDB: http://www.plantgdb.org/prj/ESTCluster/
			Glycine max	JGI Phytozome: http://www.phytozome.net/<br>PlantGDB: http://www.plantgdb.org/prj/ESTCluster/
			Glycine soja	PlantGDB: http://www.plantgdb.org/prj/ESTCluster/
			Vigna unguiculata	PlantGDB: http://www.plantgdb.org/prj/ESTCluster/
			Cajanus cajan	PlantGDB: http://www.plantgdb.org/prj/ESTCluster/
			Lotus japonicus	PlantGDB: http://www.plantgdb.org/prj/ESTCluster/
			Medicago sativa	PlantGDB: http://www.plantgdb.org/prj/ESTCluster/
			Medicago truncatula	JGI Phytozome: http://www.phytozome.net/<br>PlantGDB: http://www.plantgdb.org/prj/ESTCluster/
			Trifolium pratense	PlantGDB: http://www.plantgdb.org/prj/ESTCluster/
			Phaseolus vulgaris	PlantGDB: http://www.plantgdb.org/prj/ESTCluster/
			Phaseolus angustissimus	PlantGDB: http://www.plantgdb.org/prj/ESTCluster/
			Pisum sativum	PlantGDB: http://www.plantgdb.org/prj/ESTCluster/
			Cicer arietinum	PlantGDB: http://www.plantgdb.org/prj/ESTCluster/
			Glycyrrhiza uralensis	PlantGDB: http://www.plantgdb.org/prj/ESTCluster/
	Cucurbitales	Cucurbitaceae	*Cucumis sativus*	JGI Phytozome: http://www.phytozome.net/<br>PlantGDB: http://www.plantgdb.org/prj/ESTCluster/
	Rosales	Rosaceae	*Fragaria vesca*	PlantGDB: http://www.plantgdb.org/prj/ESTCluster/
			Malus x domestica	PlantGDB: http://www.plantgdb.org/prj/ESTCluster/
			Prunus persica	JGI Phytozome: http://www.phytozome.net/<br>PlantGDB: http://www.plantgdb.org/prj/ESTCluster/
		Cannabaceae	*Humulus lupulus*	PlantGDB: http://www.plantgdb.org/prj/ESTCluster/
			Cannabis sativa	PlantGDB: http://www.plantgdb.org/prj/ESTCluster/
	Fagales	Juglandaceae	*Juglans hindsii x Juglans regia*	PlantGDB: http://www.plantgdb.org/prj/ESTCluster/
		Fagaceae	*Quercus robur*	PlantGDB: http://www.plantgdb.org/prj/ESTCluster/
		Betulaceae	*Betula platyphylla*	PlantGDB: http://www.plantgdb.org/prj/ESTCluster/
	Malpighiales	Euphorbiaceae	*Manihot esculenta*	PlantGDB: http://www.plantgdb.org/prj/ESTCluster/
		Salicaceae	*Populus tremula x Populus tremuloides*	PlantGDB: http://www.plantgdb.org/prj/ESTCluster/
			Populus trichocarpa x Populus deltoides	PlantGDB: http://www.plantgdb.org/prj/ESTCluster/
	Vitales	Vitaceae	*Vitis vinifera*	JGI Phytozome: http://www.phytozome.net/<br>PlantGDB: http://www.plantgdb.org/prj/ESTCluster/

Supplementary material Table S2 (continued). List of species genomes of which were analyzed *in silico* in present study and the sources of the ESTs and/or genomic sequences.

Class	Order	Family	Species	Organization: Web link
Eudicotyledons	Ranunculales	Ranunculaceae	*Aquilegia formosa x Aquilegia pubescens*	PlantGDB: http://www.plantgdb.org/prj/ESTCluster/
		Papaveraceae	*Papaver somniferum*	PlantGDB: http://www.plantgdb.org/prj/ESTCluster/
	Malvales	Malvaceae	*Gossypium arboreum*	PlantGDB: http://www.plantgdb.org/prj/ESTCluster/
			Gossypium hirsutum	PlantGDB: http://www.plantgdb.org/prj/ESTCluster/
			Gossypium raimondii	PlantGDB: http://www.plantgdb.org/prj/ESTCluster/
			Theobroma cacao	PlantGDB: http://www.plantgdb.org/prj/ESTCluster/

Supplementary Material Figure S1

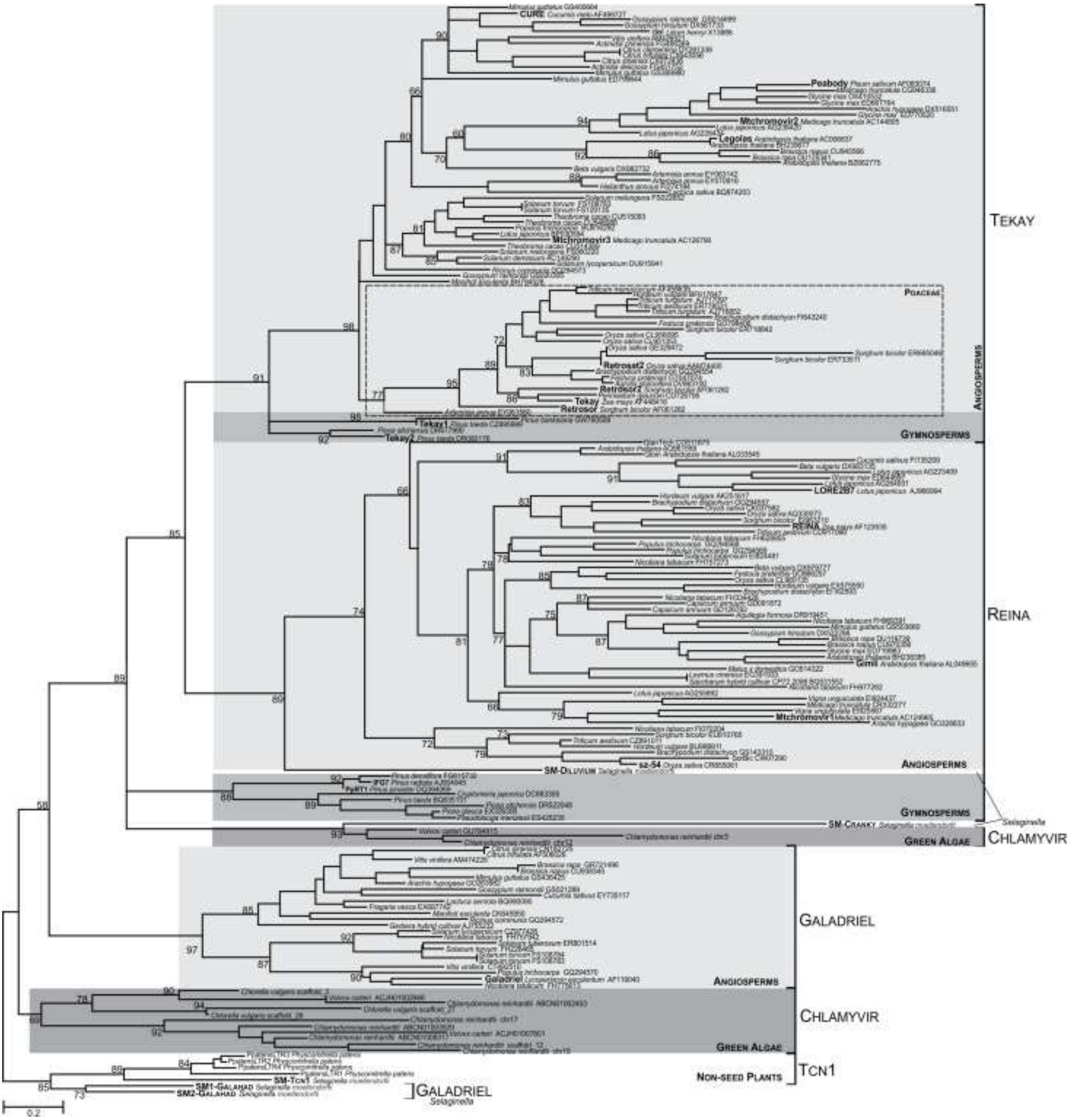

Supplementary Material Dataset S1
Analysis of CHD tree

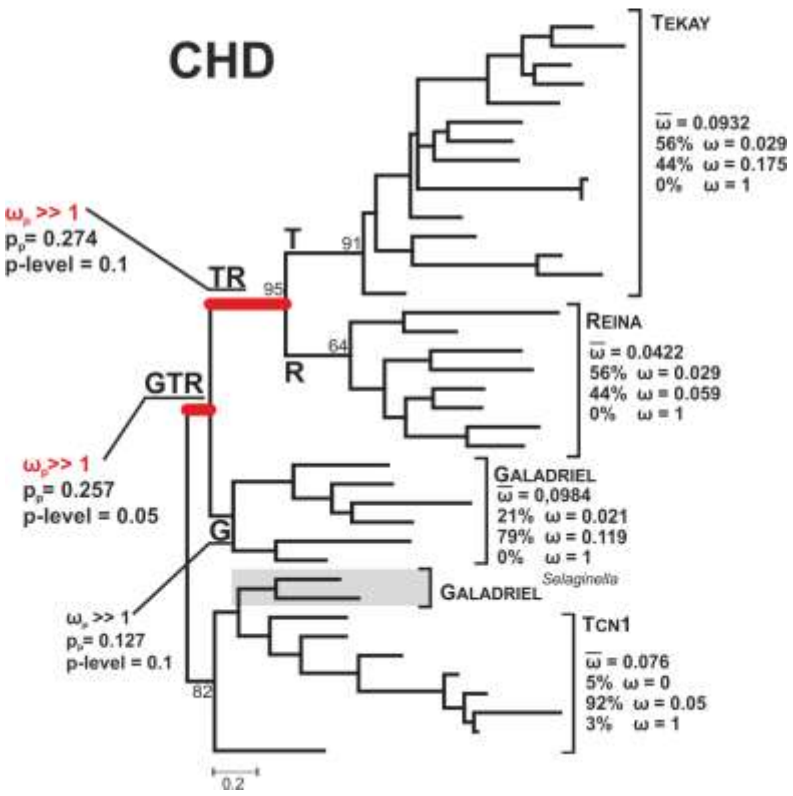

Branch-site tests.

(2.71 (0.05 level); 5.41 (0.01 level) – Null 2 vs Positive

3.84 (0.05 level); 6.63 (0.01 level) – Null 1 vs Null 2)

Branch-site test for positive selection at the base of T clade.

Model	lnL	Significant	Class 0	Class 1	Class 2a	Class 2b
Null 1 (M1a)	-5994.608301		p = 0.96436<br>ω = 0.07057	p = 0.03564<br>ω = 1.00000		
Null 2 (neutral)	-5993.232546	2.75151	p = 0.73553<br>ω_b = 0.06950<br>ω_f = 0.06950	p = 0.02732<br>ω_b = 1.00000<br>ω_f = 1.00000	p = 0.22865<br>ω_b = 0.06950<br>ω_f = 1.00000	p = 0.00849<br>ω_b = 1.00000<br>ω_f = 1.00000
Positive Selection	-5992.415185	1.634722	p = 0.71854<br>ω_b = 0.07183<br>ω_f = 0.07183	p = 0.02675<br>ω_b = 1.00000<br>ω_f = 1.00000	p = 0.24557<br>ω_b = 0.07183<br>ω_f = 999.00000	p = 0.00914<br>ω_b = 1.00000<br>ω_f = 999.00000

Naive Empirical Bayes (NEB) analysis; positive sites for foreground lineages Prob(w>1):

6 E 0.656
14 I 0.894
16 R 0.975*
24 E 0.974*
26 L 0.997**
28 K 0.900
31 G 0.592
45 L 0.651
53 E 0.954*

Bayes Empirical Bayes (BEB) analysis [73]; positive sites for foreground lineages Prob(w>1):

1 * 0.600	12 R 0.588	23 T 0.605	34 E 0.607	45 L 0.806	56 R 0.600
2 D 0.600	13 V 0.600	24 E 0.811	35 S 0.612	46 W 0.588	57 A 0.600
3 K 0.620	14 I 0.868	25 Y 0.589	36 E 0.577	47 Q 0.634	58 E 0.600
4 E 0.594	15 R 0.582	26 L 0.948	37 A 0.584	48 F 0.590	59 G 0.600
5 V 0.591	16 R 0.897	27 V 0.588	38 S 0.586	49 Q 0.595	60 A 0.600
6 E 0.783	17 R 0.590	28 K 0.785	39 W 0.588	50 E 0.591	61 T 0.600
7 I 0.602	18 G 0.611	29 W 0.588	40 E 0.575	51 Q 0.593	62 R 0.600
8 I 0.583	19 V 0.595	30 K 0.628	41 P 0.591	52 I 0.642	63 T 0.600
9 L 0.589	20 P 0.596	31 G 0.676	42 A 0.595	53 E 0.760	64 S 0.600
10 A 0.575	21 P 0.611	32 L 0.590	43 G 0.584	54 R 0.585	65 A 0.600
11 D 0.591	22 A 0.600	33 P 0.710	44 A 0.581	55 F 0.600	66 A 0.600

Branch-site test for positive selection at the base of R clade.

Model	lnL	Significant	Class 0	Class 1	Class 2a	Class 2b
Null 1 (M1a)	-5994.608301		p = 0.96436<br>ω = 0.07057	p = 0.03564<br>ω = 1.00000		
Null 2 (neutral)	-5993.469959	2.276684	p = 0.73773<br>ω_b = 0.06975<br>ω_f = 0.06975	p = 0.02736<br>ω_b = 1.00000<br>ω_f = 1.00000	p = 0.22651<br>ω_b = 0.06975<br>ω_f = 1.00000	p = 0.00840<br>ω_b = 1.00000<br>ω_f = 1.00000
Positive Selection	-5992.910964	1.11799	p = 0.69307<br>ω_b = 0.07074<br>ω_f = 0.07074	p = 0.02571<br>ω_b = 1.00000<br>ω_f = 1.00000	p = 0.27116<br>ω_b = 0.07074<br>ω_f = 19.50879	p = 0.01006<br>ω_b = 1.00000<br>ω_f = 19.50879

Naive Empirical Bayes (NEB) analysis; positive sites for foreground lineages Prob(w>1):

```
 3 K 0.885
 7 I 0.776
17 R 0.509
18 G 0.655
20 P 0.513
32 L 0.765
41 P 0.969*
42 A 0.967*
46 W 0.655
49 Q 0.557
51 Q 0.812
54 R 0.970*
55 F 0.722
```

Bayes Empirical Bayes (BEB) analysis [73]; positive sites for foreground lineages Prob(w>1):

3 K 0.945	46 W 0.792
7 I 0.619	49 Q 0.727
17 R 0.685	51 Q 0.874
18 G 0.847	53 E 0.558
20 P 0.666	54 R 0.988*
21 P 0.628	55 F 0.833
27 V 0.594	56 R 0.600
32 L 0.857	
41 P 0.990**	
42 A 0.990**	

Branch-site test for positive selection at the base of G clade.

Model	lnL	Significant	Class 0	Class 1	Class 2a	Class 2b
Null 1 (M1a)	-5994.608301		p = 0.96436<br>ω = 0.07057	p = 0.03564<br>ω = 1.00000		
Null 2 (neutral)	-5993.843327	1.529948	p = 0.83878<br>ω_b = 0.06896<br>ω_f = 0.06896	p = 0.03114<br>ω_b = 1.00000<br>ω_f = 1.00000	p = 0.12542<br>ω_b = 0.06896<br>ω_f = 1.00000	p = 0.00466<br>ω_b = 1.00000<br>ω_f = 1.00000
Positive Selection	-5991.460278	4.766098	p = 0.84189<br>ω_b = 0.07093<br>ω_f = 0.07093	p = 0.03127<br>ω_b = 1.00000<br>ω_f = 1.00000	p = 0.12229<br>ω_b = 0.07093<br>ω_f = 999.00000	p = 0.00454<br>ω_b = 1.00000<br>ω_f = 999.00000

Naive Empirical Bayes (NEB) analysis; positive sites for foreground lineages Prob(w>1):

```
21 P 0.660
46 W 0.854
48 F 0.971*
49 Q 0.923
```

Bayes Empirical Bayes (BEB) analysis [73]; positive sites for foreground lineages Prob(w>1):

```
 2 D 0.539
20 P 0.529
21 P 0.651
23 T 0.553
24 E 0.506
46 W 0.834
48 F 0.831
49 Q 0.782
51 Q 0.530
52 I 0.561
58 E 0.509
```

Branch-site test for positive selection at the base of TR clade.

Model	lnL	Significant	Class 0	Class 1	Class 2a	Class 2b
Null 1 (M1a)	-5994.608301		p = 0.96436<br>ω = 0.07057	p = 0.03564<br>ω = 1.00000		
Null 2 (neutral)	-5992.115488	4.985626	p = 0.71615<br>ω_b = 0.06855<br>ω_f = 0.06855	p = 0.02664<br>ω_b = 1.00000<br>ω_f = 1.00000	p = 0.24798<br>ω_b = 0.06855<br>ω_f = 1.00000	p = 0.00923<br>ω_b = 1.00000<br>ω_f = 1.00000
Positive Selection	-5990.255016	3.720944	p = 0.70023<br>ω_b = 0.07199<br>ω_f = 0.07199	p = 0.02601<br>ω_b = 1.00000<br>ω_f = 1.00000	p = 0.26395<br>ω_b = 0.07199<br>ω_f = 999.00000	p = 0.00981<br>ω_b = 1.00000<br>ω_f = 999.00000

Naive Empirical Bayes (NEB) analysis; positive sites for foreground lineages Prob(w>1):

```
 5 V 0.998**
11 D 0.836
25 Y 0.805
48 F 0.992**
49 Q 0.665
50 E 0.996**
52 I 0.533
53 E 0.871
```

Bayes Empirical Bayes (BEB) analysis [73]; positive sites for foreground lineages Prob(w>1):

1 * 0.599	23 T 0.667	45 L 0.566
2 D 0.599	24 E 0.726	46 W 0.589
3 K 0.695	25 Y 0.932	47 Q 0.564
4 E 0.582	26 L 0.574	48 F 0.966*
5 V 0.995**	27 V 0.548	49 Q 0.677
6 E 0.527	28 K 0.725	50 E 0.990*
7 I 0.616	29 W 0.548	51 Q 0.736
8 I 0.527	30 K 0.659	52 I 0.805
9 L 0.551	31 G 0.550	53 E 0.815
11 D 0.926	32 L 0.579	54 R 0.541
12 R 0.549	33 P 0.609	55 F 0.633
13 V 0.599	34 E 0.538	56 R 0.697
14 I 0.561	35 S 0.648	57 A 0.592
15 R 0.528	36 E 0.514	58 E 0.621
16 R 0.551	37 A 0.533	59 G 0.550
17 R 0.557	38 S 0.554	60 A 0.599
18 G 0.610	39 W 0.548	61 T 0.599
19 V 0.553	41 P 0.581	62 R 0.599
20 P 0.686	42 A 0.567	63 T 0.599
21 P 0.681	43 G 0.574	64 S 0.599
22 A 0.599	44 A 0.520	65 A 0.599
		66 A 0.599

Branch-sites test for positive selection at the base of GTR clade.

Model	lnL	Significant	Class 0	Class 1	Class 2a	Class 2b

Null 1 (M1a)	-5994.608301		p = 0.96436<br>ω = 0.07057	p = 0.03564<br>ω = 1.00000		
Null 2 (neutral)	-5992.166788	4.883026	p = 0.72582<br>ω_b = 0.06740<br>ω_f = 0.06740	p = 0.02699<br>ω_b = 1.00000<br>ω_f = 1.00000	p = 0.23832<br>ω_b = 0.06740<br>ω_f = 1.00000	p = 0.00886<br>ω_b = 1.00000<br>ω_f = 1.00000
Positive Selection	-5989.340762	5.652052	p = 0.71684<br>ω_b = 0.07097<br>ω_f = 0.07097	p = 0.02660<br>ω_b = 1.00000<br>ω_f = 1.00000	p = 0.24738<br>ω_b = 0.07097<br>ω_f = 999.00000	p = 0.00918<br>ω_b = 1.00000<br>ω_f = 999.00000

Naive Empirical Bayes (NEB) analysis; positive sites for foreground lineages Prob(w>1):

```
 3 K 0.969*
19 V 0.665
21 P 0.514
23 T 0.853
32 L 0.943
46 W 0.986*
47 Q 0.928
48 F 0.999**
49 Q 0.967*
51 Q 0.932
```

Bayes Empirical Bayes (BEB) analysis [73]; positive sites for foreground lineages Prob(w>1):

1 * 0.612	19 V 0.785	37 A 0.610	55 F 0.574
2 D 0.824	20 P 0.688	38 S 0.573	56 R 0.586
3 K 0.972*	21 P 0.696	39 W 0.572	57 A 0.697
4 E 0.530	22 A 0.612	40 E 0.528	58 E 0.623
5 V 0.569	23 T 0.814	41 P 0.572	59 G 0.612
6 E 0.533	24 E 0.549	42 A 0.588	60 A 0.612
7 I 0.624	25 Y 0.574	43 G 0.578	61 T 0.612
8 I 0.556	26 L 0.575	44 A 0.584	62 R 0.612
9 L 0.574	27 V 0.563	45 L 0.575	63 T 0.612
10 A 0.531	28 K 0.556	46 W 0.960*	64 S 0.612
11 D 0.621	29 W 0.572	47 Q 0.964*	65 A 0.612
12 R 0.574	30 K 0.547	48 F 0.996**	66 A 0.612
13 V 0.612	31 G 0.556	49 Q 0.907	
14 I 0.585	32 L 0.932	50 E 0.579	
15 R 0.577	33 P 0.627	51 Q 0.963*	
16 R 0.561	34 E 0.571	52 I 0.825	
17 R 0.584	35 S 0.572	53 E 0.603	
18 G 0.596	36 E 0.577	54 R 0.588	

Corrections for Multiple Testing

Foreground branch	base of T clade	base of R clade	base of G clade	base of TR clade	base of GTR clade
$2\Delta l$	1.634722	1.11799	4.766098	3.720944	5.652052
$p_{\chi_1^2}$	.201051	.290352	.029026	.053734	.017435
Hommel procedure $\alpha = 0.1$	TRUE	TRUE	TRUE	TRUE	**FALSE**

Hommel procedure $\alpha = 0.05$	TRUE	TRUE	TRUE	TRUE	TRUE
Hommel procedure $\alpha = 0.01$	TRUE	TRUE	TRUE	TRUE	TRUE
$p_{1/2\chi_0^2+1/2\chi_1^2}$	0.10053	0.14518	0.01451	0.02687	0.00872
Hommel procedure $\alpha = 0.1$	TRUE	TRUE	**FALSE**	**FALSE**	FALSE
Hommel procedure $\alpha = 0.05$	TRUE	TRUE	TRUE	TRUE	**FALSE**
Hommel procedure $\alpha = 0.01$	TRUE	TRUE	TRUE	TRUE	TRUE

Clade tests with several branch types (Yang's Model C).

Model	lnL	Significant	Class 0	Class 1	Class 2
Null 1 (M1a) (np=77)	-5994.608301		p = 0.96436<br>ω = 0.07057	p = 0.03564<br>ω = 1.00000	
Model C (np=84)	-5940.930269	Yes (vs Null1)	p = 0.04666<br>ω_{ALL} = 0.00000	p = 0.03336<br>ω_{ALL} = 1.00000	p = 0.91999<br>$\omega_{TCN, REINA, GALADRIEL, TEKAY}$ = 0.08036<br>ω_{GTR} = 999.00000<br>ω_{G} = 0.02933<br>ω_{TR} = 999.00000<br>ω_{T} = 999.00000<br>ω_{R} = 999.00000

Clade-sites test (Yang's Model C). (df = 3; 7.81 (0.05 level); 11.34 (0.01 level))

Clade-sites test (Yang's Model C) comparing TEKAY to REINA

Model	lnL	Significant	Class 0	Class 1	Class 2
Null 1 (M1a) (np = 46)	-3517.498312		p = 0.63068<br>ω = 0.15566	p = 0.36932<br>ω = 1.00000	
Model C (np = 49)	-3444.092199	Yes	p = 0.55513<br>ω_{TEKAY} = 0.02851<br>ω_{REINA} = 0.02851	p = 0.00000<br>ω_{TEKAY} = 1.00000<br>ω_{REINA} = 1.00000	p = 0.44487<br>ω_{TEKAY} = 0.17503<br>ω_{REINA} = 0.05868

Clade-sites test (Yang's Model C) comparing GALADRIEL to TEKAY/REINA

Model	lnL	Significant	Class 0	Class 1	Class 2
Null 1 (M1a) (np = 58)	-4631.687679		p = 0.96823<br>ω = 0.07746	p = 0.03177<br>ω = 1.00000	
Model C (np = 61)	-4567.327157	Yes	p = 0.20856<br>$\omega_{TEKAY/REINA}$ = 0.02110<br>$\omega_{GALADRIEL}$ = 0.02110	p = 0.00000<br>$\omega_{TEKAY/REINA}$ = 1.00000<br>$\omega_{GALADRIEL}$ = 1.00000	p = 0.79144<br>$\omega_{TEKAY/REINA}$ = 0.10610<br>$\omega_{GALADRIEL}$ = 0.11909

Clade-sites test (Yang's Model C) comparing TCN to GALADRIEL/TEKAY/REINA

Model	lnL	Significant	Class 0	Class 1	Class 2
Null 1 (M1a)	-5994.608301		p = 0.96436<br>ω = 0.07057	p = 0.03564<br>ω = 1.00000	
Model C (np =80)	-5940.757189	Yes	p = 0.04667<br>ω_{TCN} = 0.00000<br>$\omega_{GALADRIEL/TEKAY/REINA}$ = 0.00000	p = 0.03243<br>ω_{TCN} = 1.00000<br>$\omega_{GALADRIEL/TEKAY/REINA}$ = 1.00000	p = 0.92091<br>ω_{TCN} = 0.04987<br>$\omega_{GALADRIEL/TEKAY/REINA}$ = 0.08886

Analysis of int tree

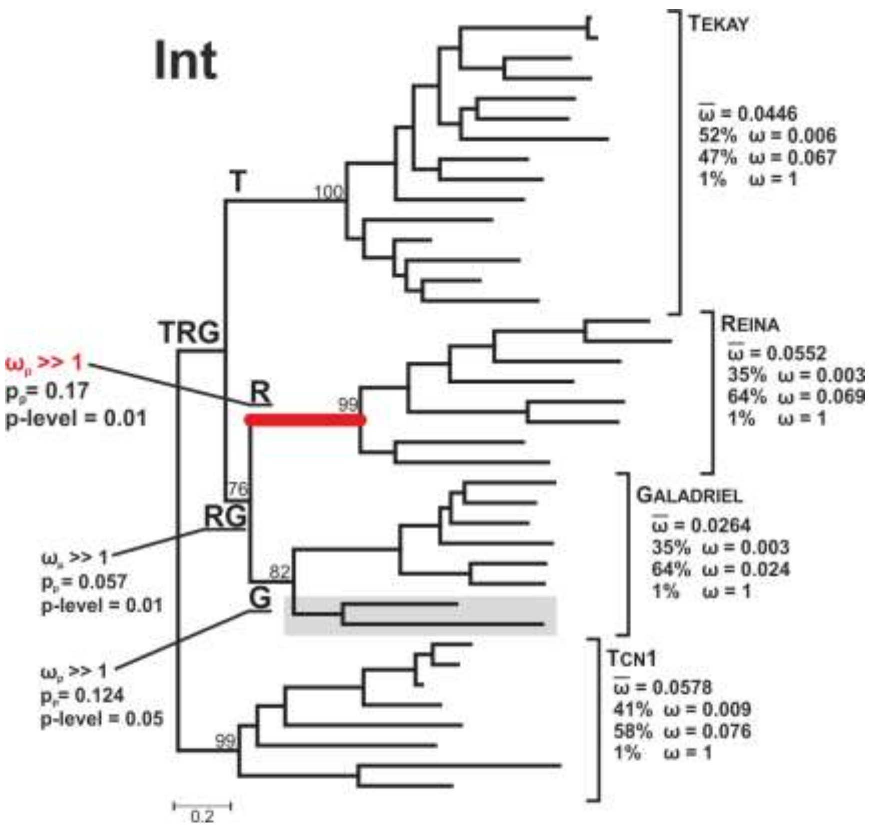

Branch-site tests.
(2.71 (0.05 level); 5.41 (0.01 level) – Null 2 vs Positive
3.84 (0.05 level); 6.63 (0.01 level) – Null 1 vs Null 2)

Branch-site test for positive selection at the base of T clade.

Model	lnL	Significant	Class 0	Class 1	Class 2a	Class 2b
Null 1 (M1a)	-13195.288420		p = 0.96147<br>ω = 0.04686	p = 0.03853<br>ω = 1.00000		
Null 2 (neutral)	-13187.570487	15.435866	p = 0.80915<br>ω_b = 0.04588<br>ω_f = 0.04588	p = 0.03326<br>ω_b = 1.00000<br>ω_f = 1.00000	p = 0.15136<br>ω_b = 0.04588<br>ω_f = 1.00000	p = 0.00622<br>ω_b = 1.00000<br>ω_f = 1.00000
Positive Selection	-13186.968977	1.20302	p = 0.83657<br>ω_b = 0.04723<br>ω_f = 0.04723	p = 0.03492<br>ω_b = 1.00000<br>ω_f = 1.00000	p = 0.12336<br>ω_b = 0.04723<br>ω_f = 2.72294	p = 0.00515<br>ω_b = 1.00000<br>ω_f = 2.72294

Naive Empirical Bayes (NEB) analysis; positive sites for foreground lineages Prob(w>1):

```
  5 V 0.978*
 22 F 0.529
 28 M 0.534
 43 L 0.573
 87 F 0.941
 89 M 0.858
118 Y 0.998**
121 C 0.977*
124 F 0.968*
127 P 0.933
133 A 0.787
```

Bayes Empirical Bayes (BEB) analysis [73]; positive sites for foreground lineages Prob(w>1):

```
  5 V 0.975*
 28 M 0.505
 43 L 0.551
 87 F 0.933
 89 M 0.842
118 Y 0.998**
121 C 0.975*
124 F 0.964*
127 P 0.920
133 A 0.785
```

Branch-site test for positive selection at the base of R clade.

Model	lnL	Significant	Class 0	Class 1	Class 2a	Class 2b
Null 1 (M1a)	-13195.288420		p = 0.96147<br>ω = 0.04686	p = 0.03853<br>ω = 1.00000		
Null 2 (neutral)	-13186.728575	17.11969	p = 0.80964<br>ω_b = 0.04324<br>ω_f = 0.04324	p = 0.03186<br>ω_b = 1.00000<br>ω_f = 1.00000	p = 0.15250<br>ω_b = 0.04324<br>ω_f = 1.00000	p = 0.00600<br>ω_b = 1.00000<br>ω_f = 1.00000
Positive Selection	-13181.218118	11.020914	p = 0.79655<br>ω_b = 0.04839<br>ω_f = 0.04839	p = 0.03335<br>ω_b = 1.00000<br>ω_f = 1.00000	p = 0.16326<br>ω_b = 0.04839<br>ω_f = 999.00000	p = 0.00684<br>ω_b = 1.00000<br>ω_f = 999.00000

Naive Empirical Bayes (NEB) analysis; positive sites for foreground lineages Prob(w>1):

```
  3 E 0.598
  8 D 0.615
 10 T 0.532
 37 Y 0.959*
 43 L 0.976*
 45 A 1.000**
 46 D 0.751
 55 A 0.998**
 69 S 0.994**
 77 V 0.999**
 80 S 0.586
 85 H 0.536
114 C 0.999**
117 M 0.990*
122 F 0.872
123 T 0.532
124 F 0.748
127 P 0.969*
129 T 0.955*
133 A 0.988*
```

Bayes Empirical Bayes (BEB) analysis [73]; positive sites for foreground lineages Prob(w>1):

```
  1 I 0.525
  2 P 0.533
  3 E 0.822
  4 Q 0.637
  5 V 0.539
  6 W 0.536
  7 D 0.683
  8 D 0.673
  9 V 0.530
 10 T 0.714
 11 M 0.505
 12 D 0.516
 13 F 0.520
 14 I 0.520
 15 T 0.558
 16 G 0.530
 17 L 0.533
```

18 P 0.533
19 L 0.556
20 S 0.534
21 * 0.558
22 F 0.541
23 G 0.530
24 F 0.531
25 T 0.519
26 V 0.558
27 I 0.519
28 M 0.537
29 V 0.525
30 V 0.533
31 V 0.525
32 D 0.516
33 R 0.538
34 L 0.526
35 S 0.532
36 K 0.521
37 Y 0.725
38 A 0.527
39 H 0.523
40 F 0.519
41 M 0.539
42 P 0.533
43 L 0.874
44 K 0.555
45 A 0.998**
46 D 0.803
47 Y 0.526
48 S 0.538
49 N 0.527
50 R 0.706
51 T 0.625
52 V 0.538
53 A 0.527
54 E 0.526
55 A 0.979*
56 F 0.520
57 V 0.566
58 N 0.558
59 Y 0.622
60 V 0.526
61 V 0.526
62 K 0.598
63 L 0.539
64 H 0.523
65 G 0.530
66 M 0.577
67 P 0.533
68 K 0.624
69 S 0.919
70 I 0.520
71 I 0.527
72 S 0.533
73 D 0.516
74 R 0.536
75 D 0.534
76 K 0.623

```
 77 V 0.976*
 78 F 0.520
 79 T 0.540
 80 S 0.629
 81 K 0.535
 82 F 0.520
 83 W 0.535
 84 Q 0.644
 85 H 0.601
 86 L 0.533
 87 F 0.520
 88 Q 0.525
 89 M 0.545
 90 Q 0.540
 91 G 0.534
 92 T 0.530
 93 T 0.522
 94 L 0.534
 95 S 0.571
 96 M 0.561
 97 S 0.525
 98 T 0.528
 99 T 0.528
100 Y 0.530
101 H 0.524
102 P 0.533
103 Q 0.531
104 T 0.528
105 D 0.516
106 G 0.531
107 Q 0.531
108 S 0.528
109 E 0.521
110 A 0.543
111 V 0.540
112 N 0.516
113 K 0.539
114 C 0.986*
115 L 0.540
116 E 0.521
117 M 0.912
118 Y 0.529
119 L 0.533
120 R 0.532
121 C 0.534
122 F 0.821
123 T 0.632
124 F 0.708
125 K 0.521
126 N 0.532
127 P 0.975*
128 K 0.577
129 T 0.932
130 W 0.535
131 F 0.547
132 K 0.551
133 A 0.935
134 L 0.533
135 T 0.533
```

136 W 0.535
137 V 0.528
138 E 0.521
139 L 0.519
140 W 0.714
141 Y 0.529
142 N 0.516

Branch-site test for positive selection at the base of G clade.

Model	lnL	Significant	Class 0	Class 1	Class 2a	Class 2b
Null 1 (M1a)	-13195.288420		p = 0.96147<br>ω = 0.04686	p = 0.03853<br>ω = 1.00000		
Null 2 (neutral)	-13191.650894	7.275052	p = 0.85179<br>ω_b = 0.04515<br>ω_f = 0.04515	p = 0.03371<br>ω_b = 1.00000<br>ω_f = 1.00000	p = 0.11014<br>ω_b = 0.04515<br>ω_f = 1.00000	p = 0.00436<br>ω_b = 1.00000<br>ω_f = 1.00000
Positive Selection	-13188.845227	5.611334	p = 0.84166<br>ω_b = 0.04762<br>ω_f = 0.04762	p = 0.03405<br>ω_b = 1.00000<br>ω_f = 1.00000	p = 0.11946<br>ω_b = 0.04762<br>ω_f = 999.00000	p = 0.00483<br>ω_b = 1.00000<br>ω_f = 999.00000

Naive Empirical Bayes (NEB) analysis; positive sites for foreground lineages Prob(w>1):

```
  8 D 0.634
 35 S 0.919
 43 L 0.761
 46 D 0.677
 57 V 0.559
 69 S 0.614
 77 V 0.715
 80 S 0.593
 84 Q 0.533
113 K 0.988*
114 C 0.669
123 T 0.913
124 F 0.569
125 K 0.685
128 K 0.671
135 T 1.000**
```

Bayes Empirical Bayes (BEB) analysis [73]; positive sites for foreground lineages Prob(w>1):

```
 35 S 0.799
 77 V 0.531
113 K 0.610
123 T 0.537
125 K 0.577
135 T 0.898
```

Branch-site test for positive selection at the base of RG clade.

Model	lnL	Significant	Class 0	Class 1	Class 2a	Class 2b
Null 1 (M1a)	-13195.288420		p = 0.96147<br>ω = 0.04686	p = 0.03853<br>ω = 1.00000		
Null 2 (neutral)	-13193.336471	3.903898	p = 0.89858<br>ω_b = 0.04498<br>ω_f = 0.04498	p = 0.03528<br>ω_b = 1.00000<br>ω_f = 1.00000	p = 0.06364<br>ω_b = 0.04498<br>ω_f = 1.00000	p = 0.00250<br>ω_b = 1.00000<br>ω_f = 1.00000
Positive Selection	-13189.089754	8.493434	p = 0.90584<br>ω_b = 0.04717<br>ω_f = 0.04717	p = 0.03729<br>ω_b = 1.00000<br>ω_f = 1.00000	p = 0.05461<br>ω_b = 0.04717<br>ω_f = 999.00000	p = 0.00225<br>ω_b = 1.00000<br>ω_f = 999.00000

Naive Empirical Bayes (NEB) analysis; positive sites for foreground lineages Prob(w>1):

```
 22 F 0.511
 37 Y 0.837
 80 S 0.999**
128 K 0.543
140 W 0.947
```

Bayes Empirical Bayes (BEB) analysis [73]; positive sites for foreground lineages Prob(w>1):

```
 75 D 0.518
 80 S 0.912
140 W 0.620
```

Branch-sites test for positive selection at the base of TRG clade.

Model	lnL	Significant	Class 0	Class 1	Class 2a	Class 2b
Null 1 (M1a)	-13195.288420		$p = 0.96147$<br>$\omega = 0.04686$	$p = 0.03853$<br>$\omega = 1.00000$		
Null 2 (neutral)	-13193.759756	3.057328	$p = 0.87726$<br>$\omega_b = 0.04592$<br>$\omega_f = 0.04592$	$p = 0.03625$<br>$\omega_b = 1.00000$<br>$\omega_f = 1.00000$	$p = 0.08306$<br>$\omega_b = 0.04592$<br>$\omega_f = 1.00000$	$p = 0.00343$<br>$\omega_b = 1.00000$<br>$\omega_f = 1.00000$
Positive Selection	-13192.774806	1.9699	$p = 0.87443$<br>$\omega_b = 0.04676$<br>$\omega_f = 0.04676$	$p = 0.03702$<br>$\omega_b = 1.00000$<br>$\omega_f = 1.00000$	$p = 0.08495$<br>$\omega_b = 0.04676$<br>$\omega_f = 75.85586$	$p = 0.00360$<br>$\omega_b = 1.00000$<br>$\omega_f = 75.85586$

Naive Empirical Bayes (NEB) analysis; positive sites for foreground lineages Prob(w>1):

```
8 D 0.945
37 Y 0.843
43 L 0.699
117 M 0.587
126 N 0.584
128 K 0.822
132 K 0.826
140 W 0.677
```

Bayes Empirical Bayes (BEB) analysis [73]; positive sites for foreground lineages Prob(w>1):

```
8 D 0.907
37 Y 0.762
43 L 0.622
117 M 0.532
126 N 0.522
128 K 0.746
132 K 0.758
140 W 0.601
```

Corrections for Multiple Testing

Foreground branch	base of T clade	base of R clade	base of G clade	base of RG clade	base of TRG clade
$2\Delta l$	1.20302	11.020914	5.611334	8.493434	1.9699
$p_{\chi_1^2}$	0.272719	0.000901	0.017845	0.003564	0.160458
Hommel procedure $\alpha = 0.1$	TRUE	FALSE	**FALSE**	FALSE	TRUE
Hommel procedure $\alpha = 0.05$	TRUE	FALSE	TRUE	**FALSE**	TRUE
Hommel procedure $\alpha = 0.01$	TRUE	**FALSE**	TRUE	TRUE	TRUE
$p_{1/2\chi_0^2+1/2\chi_1^2}$	0.13636	0.00045	0.00892	0.00178	0.08023
Hommel procedure $\alpha = 0.1$	TRUE	FALSE	FALSE	FALSE	TRUE
Hommel procedure $\alpha = 0.05$	TRUE	FALSE	**FALSE**	FALSE	TRUE
Hommel procedure $\alpha = 0.01$	TRUE	**FALSE**	TRUE	**FALSE**	TRUE

Clade tests with several branch types (Yang's Model C).

Model	lnL	Significant	Class 0	Class 1	Class 2
Null 1 (M1a) (np=77)	-13195.288420		p = 0.96147<br>ω = 0.04686	p = 0.03853<br>ω = 1.00000	
Model C (np=84)	-12812.406941	Yes	p = 0.40931<br>ω_{ALL} = 0.00947	p = 0.01283<br>ω_{ALL} = 1.00000	p = 0.57786<br>$\omega_{TCN, REINA, GALADRIEL, TEKAY}$ = 0.06924<br>ω_{TRG} = 0.12593<br>ω_{G} = 0.06454<br>ω_{RG} = 0.00010<br>ω_{T} = 0.06671<br>ω_{R} = 999.00000

Clade-sites test (Yang's Model C). (df = 3; 7.81 (0.05 level); 11.34 (0.01 level))

Clade-sites test (Yang's Model C) comparing REINA to GALADRIEL

Model	lnL	Significant	Class 0	Class 1	Class 2
Null 1 (M1a)	-5856.502483		p = 0.98481<br>ω = 0.01125	p = 0.01519<br>ω = 1.00000	
Model C (np = 36)	-5699.488755	Yes	p = 0.35473<br>$\omega_{GALADRIEL}$ = 0.00302<br>ω_{REINA} = 0.00302	p = 0.00724<br>$\omega_{GALADRIEL}$ = 1.00000<br>ω_{REINA} = 1.00000	p = 0.63803<br>$\omega_{GALADRIEL}$ = 0.02378<br>ω_{REINA} = 0.06898

Clade-sites test (Yang's Model C) comparing TEKAY to REINA/GALADRIEL

Model	lnL	Significant	Class 0	Class 1	Class 2
Null 1 (M1a)	-10538.466732		p = 0.97776<br>ω = 0.02219	p = 0.02224<br>ω = 1.00000	
Model C (np = 65)	-10221.715021	Yes	p = 0.51611<br>$\omega_{REINA/GALADRIEL}$ = 0.00616<br>ω_{TEKAY} = 0.00616	p = 0.00902<br>$\omega_{REINA/GALADRIEL}$ = 1.00000<br>ω_{TEKAY} = 1.00000	p = 0.47488<br>$\omega_{REINA/GALADRIEL}$ = 0.02813<br>ω_{TEKAY} = 0.06715

Clade-sites test (Yang's Model C) comparing TCN to TEKAY/REINA/GALADRIEL

Model	lnL	Significant	Class 0	Class 1	Class 2
Null 1 (M1a)	-13195.288420		p = 0.96147<br>ω = 0.04686	p = 0.03853<br>ω = 1.00000	
Model C (np =80)	-12819.241300	Yes	p = 0.41059<br>ω_{TCN} = 0.00934<br>$\omega_{TEKAY/REINA/GALADRIEL}$ = 0.00934	p = 0.01281<br>ω_{TCN} = 1.00000<br>$\omega_{TEKAY/REINA/GALADRIEL}$ = 1.00000	p = 0.57660<br>ω_{TCN} = 0.07643<br>$\omega_{TEKAY/REINA/GALADRIEL}$ = 0.06668

Analysis of rt tree

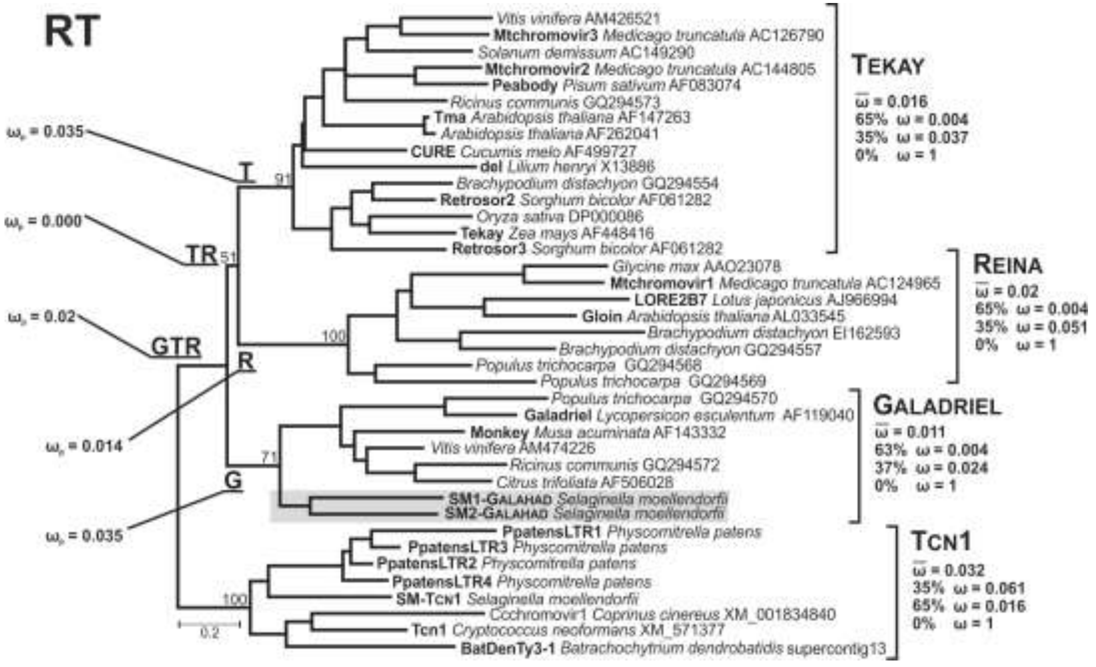

Clade tests with several branch types (Yang's Model C).

Model	lnL	Significant	Class 0	Class 1	Class 2
Null 1 (M1a) (np=77)	-12681.467466		p = 0.96420<br>ω = 0.02376	p = 0.03580<br>ω = 1.00000	
Model C (np=84)	-12301.725739	Yes	p = 0.64927<br>ω_{ALL} = 0.00898	p = 0.00000<br>ω_{ALL} = 1.00000	p = 0.35073<br>$\omega_{TCN, REINA, GALADRIEL, TEKAY}$ = 0.06463<br>ω_{TR} = 0.00010<br>ω_{G} = 0.03462<br>ω_{GTR} = 0.02032<br>ω_{T} = 0.03451<br>ω_{R} = 0.01397

Clade-sites test (Yang's Model C). (df = 3; 7.81 (0.05 level); 11.34 (0.01 level))

Clade-sites test (Yang's Model C) comparing TEKAY to REINA

Model	lnL	Significant	Class 0	Class 1	Class 2
Null 1 (M1a) (np = 46)	-7442.407249		p = 0.93598<br>ω = 0.00893	p = 0.06402<br>ω = 1.00000	
Model C (np = 49)	-7203.037533	Yes	p = 0.64828<br>ω_{TEKAY} = 0.00447<br>ω_{REINA} = 0.00447	p = 0.00000<br>ω_{TEKAY} = 1.00000<br>ω_{REINA} = 1.00000	p = 0.35172<br>ω_{TEKAY} = 0.03678<br>ω_{REINA} = 0.05055

Clade-sites test (Yang's Model C) comparing GALADRIEL to TEKAY/REINA

Model	lnL	Significant	Class 0	Class 1	Class 2
Null 1 (M1a) (np = 62)	-10080.622888		p = 0.97356<br>ω = 0.01050	p = 0.02644<br>ω = 1.00000	
Model C (np = 65)	-9754.644094	Yes	p = 0.62908<br>$\omega_{TEKAY/REINA}$ = 0.00432<br>$\omega_{GALADRIEL}$ = 0.00432	p = 0.00000<br>$\omega_{TEKAY/REINA}$ = 1.00000<br>$\omega_{GALADRIEL}$ = 1.00000	p = 0.37092<br>$\omega_{TEKAY/REINA}$ = 0.03791<br>$\omega_{GALADRIEL}$ = 0.02403

Clade-sites test (Yang's Model C) comparing TCN to GALADRIEL/TEKAY/REINA

Model	lnL	Significant	Class 0	Class 1	Class 2
Null 1 (M1a)	-12681.467466		p = 0.96420<br>ω = 0.02376	p = 0.03580<br>ω = 1.00000	
Model C (np =80)	-12305.690695	Yes	p = 0.34595<br>ω_{TCN} = 0.06136<br>$\omega_{GALADRIEL/TEKAY/REINA}$ = 0.06136	p = 0.00000<br>ω_{TCN} = 1.00000<br>$\omega_{GALADRIEL/TEKAY/REINA}$ = 1.00000	p = 0.65405<br>ω_{TCN} = 0.01572<br>$\omega_{GALADRIEL/TEKAY/REINA}$ = 0.00835
